# Comparative Investigations of Social Context-Dependent Dominance in Captive Chimpanzees (*Pan troglodytes*) and Wild Tibetan Macaques (*Macaca thibetana*)

**DOI:** 10.1038/s41598-018-32243-2

**Published:** 2018-09-17

**Authors:** Jake A. Funkhouser, Jessica A. Mayhew, Lori K. Sheeran, John B. Mulcahy, Jin-Hua Li

**Affiliations:** 1 Central Washington University, Primate Behavior Program, Ellensburg, 98926 Washington USA; 2 Washington University in St. Louis, Department of Anthropology, St. Louis, 63130 Missouri USA; 3 Central Washington University, Department of Anthropology & Museum Studies, Ellensburg, 98926 Washington USA; 4 Chimpanzee Sanctuary Northwest, Cle Elum, 98922 Washington USA; 5 Anhui University, School of Resource & Environmental Engineering, 230601, Hefei, China

## Abstract

Theoretical definitions of dominance, how dominance is structured and organized in nature, and how dominance is measured have varied as investigators seek to classify and organize social systems in gregarious species. Given the variability in behavioral measures and statistical methods used to derive dominance rankings, we conducted a comparative analysis of dominance using existing statistical techniques to analyze dominance ranks, social context-dependent dominance structures, the reliability of statistical analyses, and rank predictability of dominance structures on other social behaviors. We investigated these topics using behavioral data from captive chimpanzees (*Pan troglodytes*) and wild Tibetan macaques (*Macaca thibetana*). We used a combination of all-occurrence, focal-animal, and instantaneous scan sampling to collect social, agonistic, and associative data from both species. We analyzed our data to derive dominance ranks, test rank reliability, and assess cross-context predictability using various statistical analyses. Our results indicate context-dependent dominance and individual social roles in the captive chimpanzee group, one broadly defined dominance structure in the Tibetan macaque group, and high within-context analysis reliability but little cross-context predictability. Overall, we suggest this approach is preferable over investigations of dominance where only a few behavioral metrics and statistical analyses are utilized with little consideration of rank reliability or cross-context predictability.

## Introduction

The concept of social dominance has been adapted in models of animal behavior as a descriptive shorthand for the overall structure of complex social relationships, attributes of individuals, estimates of an individual’s power, or simply an individual’s success in agonistic competitions. While relatively few researchers agree on how dominance should be perceived^1–4^ or measured^5–10^, such structures have been found to correlate with patterns of kinship^11^, individual health^12^, reproductive success^13^, grooming received^10,14,15^, and priority access to valued resources^16–18^.

A species’, population’s, or individual’s dominance style is defined by the degree of asymmetry in agonistic relationships at the dyadic level^3^. Dominance styles are often referred to on a scale of despotic (large dyadic asymmetries and severe aggression) to egalitarian (little to no dyadic asymmetries and unresolved aggression)^19^. This scale is related to a number of social and biological variables and is most formally recognized within the *Macaca* genus^20^. The amount of social power an individual might hold over other group members is relative to the dominance style of the group. In a despotic society, a dominant individual is likely to possess more social power over a larger number of individuals than in an egalitarian society^19^. Dominance rank and status are derived to approximate any given individual’s amount of social power within a social group. An individual’s dominance rank refers to the approximated position the individual falls in the group’s social organization; these ranks are typically expressed as ordinal numbers^21^. Meanwhile, dominance status refers to an individual’s descriptive attributes and relative relationships with other individuals (e.g., dominant or subordinate), and an individual’s status is often variable between dyadic comparisons^21^.

The concept of “dominance” in animal populations originates in Schjelderupp-Ebbe’s^22^ example of a “pecking-order” in farmyard chickens. Here, dominance is operationalized as chicken A asymmetrically pecks B more than B pecks A, equating to A’s dominance over B^22^. This simple idea of directional and disproportional dyadic relationships (or dominance) has been foundational in many dominance investigations to date.

In regard to nonhuman primates, dominance is a predictive factor or common denominator across certain categories of behaviors^21,23^. However, how dominance is structured and measured (as opposed to how dominance functions) has varied as investigators seek to classify and organize the social systems of gregarious species (such as nonhuman primates). Multiple authors have argued that the applications of dominance are plagued by a lack of operational definitions, the use of multiple and various statistical analyses, ambiguous interpretations, and arbitrary meanings^2,6,21,24^. This variability has elicited debate over the *true* construct of dominance^2^. Dominance, as with many other constructs, is a lens used to describe and predict a complex network of relationships. It is the occurrence of asymmetric dyadic relationships that result in *relative* dominance statuses for each individual of a group or population. The difference in the dominance status of two individuals is one possible type of relationship that two individuals may share^21,25^. Ordinal dominance ranks depend on the behavioral context, and many studies have derived different rank orders for the same group across different measured behaviors^8,10,26–28^. Uncovering a group’s social hierarchy is most useful to predict the outcome of future interactions across multiple types of social relationships (i.e., not just the outcome of future agonistic competition). Therefore, the most appropriate categorization of a group’s dominance structure has high predictive value across other patterns of social behaviors^4,10,21^.

Dominance ranks are typically calculated in an attempt to classify, describe, and predict complex social systems in a relatively simple way (e.g., ordered ranks^21,29^). Returning to the fundamental origins of dominance, Drews^21^ operationally defines the process of measuring, categorizing, and ranking individuals that engage in dyadic *agonistic competitions* with a clearly defined “winner” and eventual submissive exchange from the “loser” to the winner. The concept of measuring dominance and deriving rank based on an individual’s observed ability to asymmetrically “win” more agonistic competitions (i.e., fights) than one “loses” is common in the nonhuman primate literature^14,17,30–34^.

Similarly, Vessey^35^ argues for dominance defined through *the lack of aggressiveness*. This context assumes individuals can predict the outcome of any given future aggressive conflict with another individual based on previous experience (e.g., displays) or encounters (e.g., fights). An individual might also recognize features in the opponent that are indicative of superiority or dominance (e.g., body/canine size)^35^. This compiled knowledge and recognition results in fleeing-upon-approach by a less dominant individual or submission/yielding when receiving threats from a more dominant individual. This dominance context has also been called “formal dominance” because dominant relationships may be readily accepted between individuals (as opposed to agonistically challenged). Therefore, conflict may be minimal and the act of submission by a subordinate formalizes the recipient’s dominant status^3^. The use of submissive, flee-upon-approach, or displacement behaviors to assess dominance is also commonly used in non-human primate investigations^14,18,36–39^.

The winning/losing or dominant/subordinate paradigm is coupled with an individual’s ability to obtain *priority access to desired resources*^40^. This method is not used to define dominance but is discussed as an attribute of an individual’s dominant position^21^. Dominance defined as access to resources does not always depend on observing aggressive competitions for immediate access to a resource (e.g., mates, food), but rather is a relatively unchallenged monopolization of a resource that others might be interested in or already possess. This dominance context has otherwise been categorized as “competitive ability,” where an individual’s success in obtaining resources (without directly winning an agonistic competition) is linked to their higher status. This type of dominance is cited in a limited number of investigations^10,40,41^.

There are also dominance definitions that focus on interactions and behaviors completely non-aggressive in nature. Wilson^42^ defines *privileged role* dominance as an animal’s privileged position in a network that represents dominance relative to other individuals. He illustrates this type of non-aggressive dominance in bees, where food is transferred from forager bees to nurses, thereby representing privilege. This exemplifies the usefulness of patterns of affiliative behavior or flow of highly desired commodities to derive such patterns of privileged role dominance. While this method is not as cited as those that refer to aggressiveness, similar measures have been used to derive dominance structures^43–45^.

Unfortunately, these various behavioral contexts for investigating dominance are not easily translated into computational methods to derive behavioral trends and individual dominance ranks. The recent literature references a disorienting variety of behavioral metrics, uses, and methods to uncover dominance structures^6,7,10^. Arguably, the field has diluted the resolution and restricted the complexity of nonhuman primate social systems to infer simple linear classifications with little predictive power^6^. Until recently, dominance was mainly investigated using David’s scores^46^, I & SI method^46^, and *h*′ tests for linearity^47^. These analyses depend on conventional matrix-based dyadic values that represent the frequency of winning over losing agonistic competitions and are largely unable to detect rank reversals or fluctuations over time. However, a few statistical analyses do provide methods to analyze dominance without underlying structural assumptions (ADAGIO^6^ and PERC^31^) or that are not matrix-based (ELO^24^). These analyses are reviewed in Methods (below).

Because of the large variation in social organization across the primate order^48^, the behavioral measures used to investigate dominance across any context should not be generalized between species, rather within species or taxa. To define these behavioral measures in our investigation of captive chimpanzees (*Pan troglodytes*) and free-ranging Tibetan macaques (*Macaca thibetana*), we drew from natural history and social organization evidence and systems or relationships that shape the dominance structures of these two species. Table 1 outlines the dominance context definitions we used for this investigation.

**Table 1:** Operational definitions for dominance and other social behavior contexts.

Species	Dominance Context	Definition
*Pan troglodytes*	Agonistic Competitions	“Winners” were simply defined as the actors of directed agonistic behaviors, where “losers” were defined as the recipient of such behaviors. These behaviors included: threat, hit/slap, bite.
	Lack of Agonism	“Winners” were defined as the recipient of submissive behaviors (pant-grunt) or actors of displacements without agonism, whereas “losers” were defined as the actors of submissive behaviors (pant-grunt) or the yielding/displaced/fleeing individual.
	Privileged Role	Because grooming is often considered a valuable commodity in chimpanzee and many nonhuman primate species, we used the directional exchange (rather than simultaneous) of grooming to quantify privileged roles. Here, the actor (groomer) was considered the “losing” individual where the recipient (groomee) was considered the “winning” individual. If individuals were observed to be grooming in polyadic fashions, directional exchanges were coded in a dyadic fashion.
	Priority Access to Resources	Due to the confines of captivity and caregiver husbandry tasks, the chimpanzees were often shifted between enclosure spaces using forage style day-time snacks or meals. We used the order entry to these newly accessible enclosure spaces to quantify individual’s priority access to resources. “Winners” were any individual who entered before any other individuals, while all other individuals were considered “losers.” This resulted in a relative number of winners and losers for all changes in access (e.g., A “beat” B, C; B “beat” C).
*Macaca thibetana*	Agonistic Competitions	“Winners” were simply defined as the actors of directed agonistic behaviors, where “losers” were defined as the recipient of such behaviors. These behaviors included: chase, threat/charge, slap/hit, grab, bite.
	Lack of Agonism	“Winners” were defined as the recipient of submissive behaviors (fear-grimace) or actors of displacements agonism, where “losers” were defined as the actors of submissive behaviors (fear-grimace) or the yielding/displaced individual (flee, retreat).
**Species**	**Other Social Behaviors**	**Definition**
*Pan troglodytes*	Affiliation	Any and all behaviors that were identified as affiliative in context. Specifically: groom, play, locomote in contact, and other affiliation as defined by AZA and JGI.
	All Agonism	Any and all behaviors that were identified as agonistic in context. Specifically: displace, hit, threat, steal object, fight, and other aggression by AZA and JGI.
	Nearest Neighbor	Any individual (chimpanzee or human) closest to the focal individual is to be recorded. More than one individual may be recorded if multiple individuals are within equal distance of the focal. The focal is said to be without any neighbors if the focal is not engaged in an interaction with any other(s) and no individuals are within 10 ft of the focal.
*Macaca thibetana*	Grooming	Picking through hair or at skin of another individual and removing debris with hands and/or mouth. During simultaneous grooming, both individuals are to be recorded as “actors” of this behavior with the other coded as “recipient.”
	Maternal Kinship	While it is clear that maternal relatedness is not a social behavior, since many aspects of social life for Tibetan macaques are kin-biased and for ease of explanation we consider it under this category hereafter.

AZA refers to Association of Zoos and Aquariums^92^ and JGI refers to Jane Goodall Institute^93^. All polyadic interactions were coded in a dyadic fashion (e.g., A:B, B:C, A:C).

Wild chimpanzees inhabit wide ranges across equatorial Africa and thrive in large multi-male/multi-female fission-fusion societies of 22 to over 140 individuals. To ensure access to adequate resources, individuals within these large communities must navigate dynamic dominance hierarchies and complicated social structures^49,50^. Male chimpanzees typically adhere to strict, linear hierarchies as part of frequent competition in accessing estrous females and other valuable resources^51^. All males are often dominant over all females, and therefore, most non-competitive male aggression is female-focused^52,53^. Female rank is correlated with priority access to preferred food resources, reproductive success, and infant survival^16,54^. During, or immediately following, aggressive encounters, chimpanzees seek or offer reassurances by/from those with whom they share strong social bonds^49^. Following agonistic encounters, two or more combatants may reconcile with affiliative behaviors to rebuild social bonds, mend relationships, and decrease the probability of future aggression^53^. Although this is generally true of wild chimpanzee relations, some authors have observed female chimpanzees form stable relationships, share food, and form coalitions to counter male aggression in both captive and wild groups^18,55^. Captive chimpanzees have been observed to engage in aggression much less frequently than their wild counterparts, possibly as a strategy to limit stress or as a byproduct of increased tolerance in the confines of captivity^56–59^.

Dominant chimpanzees occupy central positions in grooming networks; specifically, Kanngiesser, Sueur, Riedl, Grossmann, and Call^60^ found that grooming eigenvector centrality was correlated with high dominance rank (derived through directional agonism). Relationships have been discovered between various types of dominance interactions and grooming rates/partners in wild and captive chimpanzees and bonobos^10,14^. In general, grooming across many nonhuman primate species, including chimpanzees, is considered a keystone metric for social bonds^61–64^, status^14,65^, and even a commodity (an interchangeable or exchangeable good; e.g., grooming exchanged for grooming, or grooming for agonistic support, social tolerance, and/or valued food)^32,66^. Seyfarth^15^ posits that dominant individuals are “attractive” grooming partners on the basis of building support for future agonistic interactions. Thus, previous investigations predict that primates focus grooming towards higher ranking individuals or with those with whom they share strong social bonds. Therefore, it follows that unknown dominance relationships might be accurately gauged through measuring group-wide patterns of directional grooming. Overall, it becomes clear that chimpanzees have a complex dominance structure that can be agonistically challenged^17,53,55^, conveyed through submissive behaviors^14,18^, exploited for access to resources^51^, and predicted through directional allogrooming^14,15,62,67^.

Tibetan macaques (*Macaca thibetana*) live in multi-male, multi-female groups of 15–50 individuals, and the sex ratios of these groups often favor females^20,30^. As is common across many macaque species, Tibetan macaque males disperse as they reach sexual maturity (>8 years) and can continue to transfer between groups throughout their lifespan^20,68^. Adult males are commonly thought to be the highest-ranking individuals, although females have been found to outrank some males^30^. The social organization of Tibetan macaques is a strictly linear dominance style and strong kin biases and coalitions^30^. The dominance rank of females is considered matrilineal; females have been found to attain the rank below their mother but above their older siblings^30,68,69^. Thierry *et al*.^20^ found Tibetan macaques best resembled a grade-three species on the Macaque Dominance Style Grade Scale. However, the species was later elevated to a grade-two despotic species^30^. These authors constructed dominance hierarchies using directional submissive interactions and found that agonism occurred at rates similar to despotic macaque species, but conciliatory tendencies were lower (especially between female dyads)^30^.

Tibetan macaques groom at symmetric rates (exchange grooming for grooming received), prefer female kin grooming partners, and females prefer to groom higher-ranking females (even if unrelated)^70^. Xia *et al*.^71^ also found positive correlations between dyadic tolerance and the frequency/duration of lower-ranking males grooming higher-ranking males. These investigations illuminate the generally despotic nature of Tibetan macaque social organization^20,30^, bias for female kin across a number of social contexts (namely, coalitionary support, grooming, and infant handling^30^), and the overall value of grooming in this species^70,71^.

A number of studies have utilized any one, a combination, or manipulated variations of these behavioral contexts and statistical methods to derive hierarchical dominance ranks for captive and wild nonhuman primate populations. With current and past variability in the behavioral measures and statistical methods used to derive dominance, we propose an approach that utilizes existing statistical techniques to analyze dominance ranks, social context-dependent dominance, and the reliability of statistical analyses. Specifically, in this investigation we aimed to (1) explore the use of a comparative approach to the statistical analyses of dominance hierarchies with captive chimpanzees and validate such an approach with Tibetan macaque data, (2) test the reliability of ranking orders within and across dominance contexts, and (3) test the cross-context predictability of these derived dominance structures on other social behaviors. To do so, we investigated a group of captive chimpanzees (*Pan troglodytes*) and validated our approach with archival data from wild Tibetan macaques (*Macaca thibetana*). Because various methodological and statistical techniques used to derive rank are used across the nonhuman primate literature (regardless of taxa, social organization, group composition, group size, wild or captive settings, etc.), we wanted to explore the utility of our comparative approach with data sets that reflect such diversity to some degree. Specifically, we examined dominance hierarchies using a number of behavioral contexts (e.g., agonistic competitions, lack of agonism, privileged role, and priority access to resources) and statistical techniques (DS, I&SI, ELO, ADAGIO, and PERC). We investigated the reliability of these derived ranks within each dominance context (across statistical tests) and across dominance contexts using individual median rank calculations and Spearman’s *rho* correlation coefficients in SPSS^72^. We also investigated the cross-context predictability of these derived hierarchies on the social networks of other social measures (affiliative, grooming, and nearest-neighbor networks) using MR-QAP multiple matrix correlation/regression analyses in UCINET^73^.

## Results

During all-occurrence sampling at CSNW with the captive chimpanzee group, J.A.F. recorded a total of 2294 agonistic events, 1263 affiliative events, and 62 changes in access. J.A.F. also collected 517 instantaneous scan samples (at 20:33 ± 49:57 minutes apart) where all individuals were recorded to have 0.9 ± 0.6 nearest neighbors per scan (roughly 1 ± 1 nearest neighbors). During all-occurrence sampling at Mt. Huangshan with the wild adult Tibetan macaque group, L.K.S. recorded 414 agonistic events, and J.A.M. recorded 646 grooming bouts during focal-follows. Study group demographics are reported in Table 2. Interaction matrices for all contexts are reported in Supplementary Tables.

**Table 2:** Demographic of study groups.

Name	Abbreviation	Species	Sex	Estimated Age*
Annie	Ann	*Pan troglodytes*	M	42
Burrito	Bur	*Pan troglodytes*	M	33
Foxie	Fox	*Pan troglodytes*	F	40
Jamie	Jam	*Pan troglodytes*	F	38
Jody	Jod	*Pan troglodytes*	F	41
Missy	Mis	*Pan troglodytes*	F	41
Negra	Neg	*Pan troglodytes*	F	43
BaiTou	BT	*M. thibetana*	M	26
DuanShou	DS	*M. thibetana*	M	14
GouShan	GS	*M. thibetana*	M	31
HuaHong	HH	*M. thibetana*	F	13
HuangMa	HM	*M. thibetana*	M	14
HeiTou	HT	*M. thibetana*	M	24
HuaXiaMing	HXM	*M. thibetana*	M	6
TouGui	TG	*M. thibetana*	M	13
TouHong	TH	*M. thibetana*	F	13
TouHuaYu	THY	*M. thibetana*	F	7
TouRui	TR	*M. thibetana*	F	12
TouRongGang	TRG	*M. thibetana*	M	6
TouRongYu	TRY	*M. thibetana*	F	7
TouTai	TT	*M. thibetana*	F	25
TouHuaXue	TXH	*M. thibetana*	F	7
TouXiaXue	TXX	*M. thibetana*	F	8
YeChunYu	YCY	*M. thibetana*	F	7
YeHong	YH	*M. thibetana*	F	13
YeMai	YM	*M. thibetana*	F	26
YeRongBing	YRB	*M. thibetana*	M	8
YeRongQiang	YRQ	*M. thibetana*	M	6
YeXiaXue	YXX	*M. thibetana*	F	6
YeZhen	YZ	*M. thibetana*	F	24
ZouBa	ZB	*M. thibetana*	M	14

This table details individual identities, abbreviations, species, sex, & age (in years). *Chimpanzee age estimates are from 2017 and Tibetan macaque age estimates are from 2016. Age estimates were collected from site managers (Directors of CSNW and Ahnui University researchers) and are reported in years.

Because we used a large number of statistical analyses in this investigation, we report the majority of our statistical results in formatted tables (Tables 3–8). Table 3 contains an abbreviated summary of the results that follow. Tables 4 and 5 detail all rank results for captive chimpanzees (Table 4) and adult Tibetan macaques (Table 5) across all ranking procedures and all dominance contexts. Table 6 contains Spearman’s rank correlation coefficient results for pair-wise rank order comparisons across rank analyses but within dominance contexts. Many of the pair-wise comparisons between ranking methods were significant (P < 0.05); all derived ranks for the Tibetan macaque group were significantly correlated, while most ranks for the chimpanzee group were significantly correlated (most correlated: lack of agonism; least correlated: agonistic competitions). Generally, these results indicate that there is high reliability between dominance ranking statistics when compared within dominance contexts. Table 7 contains Spearman’s rank correlation coefficient results for comparisons between median ranks for each dominance context against other dominance contexts. Notably, for chimpanzee median ranks only lack of agonism and access to resources ranks were significantly correlated (R = 0.766, P = 0.04). For Tibetan macaques, agonistic competition and lack of agonism ranks were significantly correlated (R = 0.877, P < 0.01). Of these seven pair-wise comparisons, only two were statistically significant, and the lack of correlations indicates that there is low reliability between the four dominance contexts for chimpanzees, but high reliability between the two Tibetan macaque dominance contexts. Table 8 contains MR-QAP correlation and regression results for differences in median dominance ranks (between dyads) for each dominance context against other social behaviors. MR-QAP regression analyses were only calculated for context correlations that were found to be statistically significant (P < 0.05). Most interestingly, only three of the 12 pair-wise comparisons resulted in significant correlations for the chimpanzee group: lack of agonism/all agonism (R = −0.444, P = 0.02; Y = −0.036X + 0.86; R^2^ = 19.7%), access to resources/affiliation (R = 0.186, P = 0.01; Y = 0.012X −0.62; R^2^ = 3.3%), and privileged role/affiliation (R = 0.185, P < 0.01; Y = 0.011X −0.639; R^2^ = 3.4%). For the Tibetan macaque data, only lack of agonism correlated with maternal relatedness (R = 0.427, P < 0.01; Y = 0.262X −19.09; R^2^ = 20.4%). Supplementary Figs 1-18 provide *graphic outputs* that diagram interactions from ADAGIO and Elo-rating procedures and dominance certainty heat-maps from PERC procedures.

**Table 3:** Summary of methods and results.

Species	Study Site	Data Collection Technique	Dominance Contexts	Linearity, Stability, & Null Dyads	Rank Analyses	Correlations with other Social Behaviors
Captive Chimpanzees (*Pan troglodytes*, N = 7)	Chimpanzee Sanctuary Northwest (Cle Elum, WA, USA)	All-occurrence agonistic and affiliative data collection three days per week from June through August 2017 (N* = *31)	Agonistic Competition	*h*′ = 0.50P = 0.256 Stab = 0.92 Null = 0	DSˆ	Affiliation Agonism Nearest Neighbor
					I & SIˆ	
					ELO	
					PERCˆ	
					ADAGIOˆ	
			Lack of Agonism^§^	*h*′ = 0.93* P < 0.01 Stab = 0.98 Null = 0	DSˆ	Affiliation Agonism^◊^ Nearest Neighbor
					I & SIˆ	
					ELOˆ	
					PERCˆ	
					ADAGIOˆ	
			Privileged Role	*h*′ = 0.93* P < 0.01 Stab = 0.98 Null = 0	DSˆ	Affiliation^◊^ Agonism Nearest Neighbor
					I & SIˆ	
					ELOˆ	
					PERCˆ	
					ADAGIOˆ	
			Priority Access to Resources^§^	*h*′ = 0.75* P = 0.04 Stab = 0.91 Null = 0	DSˆ	Affiliation^◊^ Agonism Nearest Neighbor
					I & SIˆ	
					ELO	
					PERCˆ	
					ADAGIOˆ	
Free-ranging (provisioned) adult Tibetan macaques *(M. thibetana*, N* = *24)	Mt. Huangshan (Anhui Province, China)	All-occurrence agonistic and focal-follow allogrooming data collection daily from July through August 2016 (N* = *36)	Agonistic Competition^§^	*h*′ = 0.34* P < 0.001 Stab = 0.99 Null = 60%	DSˆ	Grooming Maternal Kinship
					I & SIˆ	
					ELOˆ	
					PERCˆ	
					ADAGIOˆ	
			Lack of Agonism^§^	*h*′ = 0.10 P = 0.06 Stab = 0.99 Null = 59%	DSˆ	Grooming Maternal Kinship^◊^
					I & SIˆ	
					ELOˆ	
					PERCˆ	
					ADAGIOˆ	

Notably, in the chimpanzee group, dominance interactions were only significantly linear (*h*′ > 0.90, P < 0.05) for lack of agonism and privileged role contexts. These same contexts had the highest rank stability (stab >0.98). In the Tibetan macaque group, neither dominance context was considered linear: although this test was statistically significant (P < 0.01), neither *h*′ value exceeded 0.90 (specifically, *h*′ <0.35). However, interactions in both contexts were found to be highly stable (stab >0.99). Section marks (^§^) denote statistically significant correlations (P < 0.05) between median dominance rank for each context within species. Asterisks (*) denote statistically significant results (P < 0.05) for tests of linearity. Circumflexes (ˆ) denote statistically significant correlations (P < 0.05) between ranking statistics within dominance contexts. Diamonds (^◊^) denote statistically significant correlations (P < 0.05) between median dominance context ranks and other behavioral contexts. “Stab” refers to Elo dominance stability and “null” refers to the percentage of null dyads for each context.

**Table 4:** Detailed rank results for all captive chimpanzees across all statistical tests (DS, I&SI, Elo, ADAGIO, PERC, and median rank) within each dominance context (agonistic competition, lack of agonism, privileged role, access to resources).

ID	Median Rank	DS	I & SI	ELO	ADAGIO	PERC
**Agonistic Competitions**						
Jamie	*1*	1	1	3	1	1
Foxie	*2*	4	2	1	2	2
Burrito	*3*	2	4	2	3	3
Missy	*4*	3	4	4	2	5
Negra	*4*	5	4	6	4	4
Jody	*6*	6	6	7	4	6
Annie	*7*	7	7	5	5	7
		*	*		*	*
**Lack of Agonism**						
Burrito	*1*	1	1	1	1	1
Jamie	*2*	2	2	2	2	2
Negra	*3*	3	3	3	3	3
Jody	*4*	4	4	4	4	4
Foxie	*6*	5	6	6	6	5
Missy	*6*	6	6	5	5	6
Annie	*7*	7	6	7	6	7
		*	*	*	*	*
**Privileged Role**						
Negra	*1*	1	1	1	1	1
Foxie	*2*	2	2	3	2	2
Jody	*3*	3	3	2	3	3
Jamie	*4*	4	4	4	4	4
Burrito	*5*	5	7	5	5	5
Missy	*6*	6	5	6	6	6
Annie	*7*	7	6	7	7	7
		*	*	*	*	*
**Priority Access to Resources**						
Jamie	*1*	1	1	1	1	1
Negra	*2*	2	2	4	2	4
Foxie	*3*	4	3	5	3	2
Burrito	*3*	3	5	3	5	3
Jody	*4*	5	4	2	4	6
Missy	*6*	6	6	6	6	5
Annie	*7*	7	7	7	7	7
		*	*		*	*

Asterisks (*) denote significant correlations (P < 0.05) within dominance contexts.

**Table 5:** Detailed rank results for adult Tibetan macaques across all ranking procedures (DS, I&SI, Elo, ADAGIO, PERC, and median rank) within both dominance contexts (agonistic competitions and lack of agonism).

Agonistic Competition Rank Results							Lack of Agonism Rank Results						
Monkey ID	Median Rank	DS	I&SI	ADAGIO	PERC	ELO	Monkey ID	Median Rank	DS	I&SI	ADAGIO	PERC	ELO
TG	2	1	2	2	6	1	TG	1	1	1	1	6	1
YRB	2	2	1	1	5	2	YRB	2	3	2	2	4	2
GS	3	3	3	2	7	8	GS	3	4	3	3	7	3
BT	4	4	4	3	8	3	YH	4	5	4	3	10	4
TR	5	7	6	5	4	4	YXX	4.5	2	5	4	11	6
TXH	5	5	5	6	1	6	BT	5.5	7	6	4	9	5
YCY	6	6	7.5	3	9	5	TR	6.5	8	8	6	1	7
TRY	7	8	7.5	5	3	7	YCY	6.5	6	7	6	8	14
TRG	9	12	13	7	2	9	TRG	8.5	12	13.5	6	3	11
HH	10	10	12.5	6	16	10	HH	9	9	11	6	16	9
YH	10.5	14	10.5	4	10	17	ZB	9	16	10	6	13	8
YXX	11	9	11.5	5	11	13	TXH	10	15	16	5	5	16
ZB	11	11	9	5	13	12	HM	10.25	11	10.5	6	21	10
TXX	14	13	14.5	7	14	19	TH	11.5	10	15	5	18	13
YM	14	15	16.5	8	12	14	TRY	11.5	17	17	6	2	20
DS	15.5	16	15.5	6	15	18	YM	12	13	12	5	12	12
HM	17	17	18.5	7	21	16	HT	13	14	12	6	17	15
HT	17	19	20	7	17	11	DS	16	18	20	7	15	17
TH	18	20	17.5	7	18	20	TXX	16	19	19	5	14	18
YRQ	18	18	15	8	20	22	YRQ	19.5	20	21	7	20	19
HXM	19	23	23	8	19	15	HXM	20	21	22	7	19	21
YZ	21	21	21	8	24	23	THY	20	22	18	7	22	23
THY	22	24	24	8	22	21	TT	22.5	24	24	7	23	22
TT	22	22	22	8	23	24	YZ	23	23	23	7	24	24
		*	*	*	*	*			*	*	*	*	*

Asterisks (*) denote significant correlations (P < 0.05) within dominance contexts.

**Table 6:** Detailed Spearman’s rank correlation coefficient results for pair-wise rank order comparisons across rank analyses but within dominance contexts for both the captive chimpanzee and wild Tibetan macaque groups.

Rank Statistic	Rank Statistic	Captive Chimpanzees								Wild Tibetan Macaques			
		Agonistic Competitions		Lack of Agonism		Access to Resources		Privileged Role		Agonistic Competition		Lack of Agonism	
		R	P	R	P	R	P	R	P	R	P	R	P
DS	I&SI	0.815	0.02	0.964	<0.01*	0.893	<0.01*	0.893	<0.01*	0.975	<0.01*	0.947	<0.01*
DS	Elo	0.631	0.13	0.964	<0.01*	0.750	0.06	0.964	<0.01*	0.88	<0.01*	0.920	<0.01*
DS	ADAGIO	0.873	0.01	0.937	<0.01*	0.893	<0.01*	1.00	<0.01*	0.884	<0.01*	0.826	<0.01*
DS	PERC	0.821	0.02	1.00	<0.01*	0.821	0.02	1.00	<0.01*	0.846	<0.01*	0.619	<0.01*
I&SI	Elo	0.667	0.10	0.964	<0.01*	0.714	0.09	0.857	0.14	0.833	<0.01*	0.943	<0.01*
I&SI	ADAGIO	0.906	<0.01*	0.972	<0.01*	1.00	<0.01*	0.893	<0.01*	0.904	<0.01*	0.812	<0.01*
I&SI	PERC	0.064	<0.01*	0.964	<0.01*	0.750	0.05	0.893	<0.01*	0.846	<0.01*	0.603	<0.01*
Elo	ADAGIO	0.655	0.11	0.991	<0.01*	0.714	0.09	0.964	<0.01*	0.782	<0.01*	0.818	<0.01*
Elo	PERC	0.714	0.07	0.964	<0.01*	0.535	0.24	0.964	<0.01*	0.834	<0.01*	0.585	<0.01*
ADAGIO	PERC	0.818	0.02	0.937	<0.01*	0.750	0.05	1.00	<0.01*	0.71	<0.01*	0.599	<0.01*

“R” indicates the correlation coefficient, “P” indicates the corresponding P-value. Asterisks (*) denote statistically significant (P < 0.01) correlations which was reduced to minimize chance of Type 1 error.

**Table 7:** Detailed Spearman’s rank correlation coefficient results for *comparisons between median ranks for each dominance context* against other dominance contexts for both species (e.g., median agonistic competition ranks vs. median lack of agonism ranks).

Species	Dominance Context	Dominance Contexts	Correlation	
			R	P
Captive Chimpanzees	Agonistic Competition	Lack of Agonism	0.555	0.20
	Agonistic Competition	Access to Resources	0.763	0.05
	Agonistic Competition	Privileged Role	0.342	0.45
	Lack of Agonism	Access to Resources	**0.766**	0.04*
	Lack of Agonism	Privileged Role	0.324	0.48
	Access to Resources	Privileged Role	0.721	0.07
Wild Tibetan Macaques	Agonistic Competition	Lack of Agonism	**0.877**	<0.01*

“R” indicates the correlation coefficient, “P” indicates the corresponding P-value. Asterisks (*) denote statistically significant (P < 0.05) correlations.

**Table 8:** Detailed MR-QAP correlation and regression results for differences in median dominance ranks (between dyads) for each dominance context against other social behaviors (chimpanzees: all directional affiliation, all directional agonism, nearest neighbor associations; Tibetan macaques: directional grooming and maternal kinship relatedness).

Dominance Context	Cross-Context	Correlation		Regression		
		R	P	β	α	R^2^
Agonistic Competitions	All Agonism	−0.195	0.24			
	Affiliation	0.136	0.10			
	Nearest Neighbor	0	0.38			
Lack of Agonism	All Agonism	**−0.444**	0.02*	−0.036	0.860	0.197
	Affiliation	0.12	0.10			
	Nearest Neighbor	0	0.38			
Access to Resources	All Agonism	−0.187	0.26			
	Affiliation	**0.186**	0.01*	0.012	−0.620	0.033
	Nearest Neighbor	0	0.43			
Privileged Role	All Agonism	0.102	0.38			
	Affiliation	**0.185**	<0.01*	0.011	−0.639	0.034
	Nearest Neighbor	0	0.44			
**Wild Tibetan Macaques**						
Agonistic Competition	Grooming	−0.018	0.43			
	Kinship	0.221	0.12			
Lack of Agonism	Grooming	0.111	0.14			
	Kinship	**0.427**	<0.01*	0.262	−19.091	0.204

“R” indicates the regression correlation coefficient, “P” indicates the corresponding P-value, “β” indicates the beta value (slope of regression equation), “α” indicates the alpha value (y-intercept of regression equation). Asterisks (*) denote statistically significant (P < 0.05) results. It is important to note that in the investigations of captive chimpanzees, lack of agonism and all agonism are not independent samples nor are privileged role and affiliation; therefore, these significant correlation and regression results are not as impactful as the other significant results in this table.

## Discussion

Our statistical analyses revealed that many derived ranking orders correlated between ranking statistics within dominance contexts (high rank-order reliability), however, few median rank orders correlated between dominance contexts or across social networks (context-dependent predictability). Because of the lack of reliability or predictability between dominance statistics and derived ranks of either species, these results indicate that careful consideration is imperative when collecting dominance interaction data, choosing ranking procedures, and interpreting dominance results. Our conclusions support other theoretical and applied investigations of dominance and illuminate the need for further investigations of context-dependent dominance while urging caution in constructing complex agonistic networks to a single linear ranking order. We acknowledge that this comparative approach is time-consuming and requires different types of behavioral data, but it is effective at providing a holistic lens for depicting dominance relationships in nonhuman primate groups. It may also more accurately reflect how nonhuman primates use dominance relationships in fluid, situation-specific ways in the ever-changing social groups they live in.

This research contributes to conversations of context-dependent dominance in nonhuman primate societies. Our analyses of chimpanzee dominance in this investigation derived four independent rank orders indicating little cross-context dominance rank reliability. Similar results of context-dependent dominance have been found by Noė *et al*.^27^ with captive chimpanzees, Vervaecke *et al*.^10^ with captive bonobos, and others (*Saimiri sciureus*^1^, *Macaca mulatta*^74^, *Lemur catta*, *Eulmer rufus-collaris* hybrid^8^, *Propithecus verreaxi*^4,8^). Analyzing dominance hierarchies using different behavioral measures is useful to differentiate individual social roles and holistically depict the specific nature of dyadic relationships. For example, in the captive chimpanzee group at CSNW it becomes clear that Jamie engages in a relatively large number of asymmetric agonistic interactions (agonistic competition dominance), and she obtains priority access to desired resources (priority access dominance). Similarly, all individuals submit or yield to Burrito’s aggression in a formalized fashion (lack of agonism dominance). However, neither Jamie nor Burrito holds a privileged role in the group, as Negra receives a large amount of asymmetric grooming (privileged role dominance). Previously, we investigated the social relationships within this captive chimpanzee group and were unable to generate such detailed descriptions of individual roles because of their simplified investigation of dominance^56^.

The lack of significant dominance linearity in agonistic competition ranks in the chimpanzee group is interesting considering that Noė *et al*.^27^ argues that the most useful dominance ranks are linear, steep, and consistent in describing the interactions between individuals. With these criteria, it is unclear if agonistic competition dominance has relevance in the captive chimpanzee populations. Further evidence supporting this notion is the lack of correlation between agonistic competition and any other social behavior. Funkhouser, Mayhew, and Mulcahy^56^ attributed Jamie’s dominant position to her aggressive tendencies and temperament rather than a mutually agreed upon (or formal) dominant position (evident through her isolated position in affiliative networks). The current evidence supports this claim as Burrito and Negra were found to be dominant in contexts that better depict formal, perceived, or respected dominant positions accompanied by greater social power^19,27,74,75^. These claims further discount the likely impact of our derived agonistic competition dominance on the social relationships and underlying structure of the CSNW chimpanzee group. The nuances between our dominance rank results provides a more holistic description of this group’s overall dominance structure than would have been possible with only one lens of dominance (e.g., agonism^56^).

Our derived dominance structures for the chimpanzee group did not broadly correlate with other social behaviors. The negative correlations between lack of agonism dominance and all agonism is most likely due to natural patterns of behaviors: those that emit a lot of aggression (e.g., Jamie & Burrito) are not submitting or yielding to others’ aggression^10,27^. Similarly, the correlation between differences in privileged role rank and affiliation can be largely attributed to the nature of social bonds: the directionality of grooming corresponds with the occurrence of other affiliative behaviors (including grooming itself)^63,75^. Most interestingly, the significant correlation between access to resource dominance and affiliation corresponds with other evidence that suggests ties between patterns of affiliation, reconciliation, coalitionary support, and competitive ability^14,18,60,76^. This correlation coupled with the results of other authors might imply that the chimpanzees are using affiliation in non-aggressive contexts to mediate their close interactions immediately prior to obtaining access to priority resources.

The relaxed dominance style, complex social system, and behavioral flexibility of chimpanzees might allow for such individualized dominant roles and the ecological validity of context-dependent dominance in a captive setting. Further, the nature of static group membership (high familiarity and implied stability) and captive constraints (high spatial density) might further enforce these flexible and context-dependent structures in sanctuary-living chimpanzees. Funkhouser, Mayhew, and Mulcahy^56^ and Nieuwenhuijsen and de Waal^58^ noted that collecting substantial amounts of agonistic interactions in captive chimpanzees is challenging. Vervaecke *et al*.^10^ describe the benefit of context-dependent dominance investigations in situations where agonism is infrequent. We posit that utilizing all-occurrence sampling methods in captive settings increases the opportunity for observing such behaviors in investigations of dominance.

In validating this approach, the Tibetan macaque dominance analyses revealed high reliability between dominance contexts; however, only differences in lack of agonism ranks correlated with maternal relatedness. These results indicate that our investigations yield one, broadly defined hierarchy that is not context-dependent. This result largely corresponds with research by Berman, Ionica, and Li^30^ defining Tibetan macaques as a grade 2 (despotic) species. The moderate asymmetric patterns of aggression, low conciliatory tendencies, and little tolerance around resources presumes that many aspects of social interactions are highly moderated by a universally accepted individual dominance status^20,69^. Because of these trends, we believe our results indicating a broadly defined and universal hierarchy in this group fits well with the expectations of Tibetan macaque dominance style^20,69^. However, we collected far fewer agonistic data on the Tibetan macaque group than the chimpanzee group; it is also possible that the percent of dyads that were not observed to interact (≈60% unknown relationships) impacted these results by increasing their similarity^7^. In any case, our comparative approach of calculating individual median ranks across different ranking procedures minimizes the error and takes conservative interpretations of dominance with minimal data.

The large proportion of unknown relationships between dyads might have also contributed to our results of few cross-context correlations between dominance and other social behaviors in the Tibetan macaque group. Our results only indicate a significant correlation between the difference in a lack of agonism status and maternal relatedness (kinship) of this group (i.e., small differences in rank correlated with increased relatedness). The dominance rank of Tibetan macaques (specifically females) is maternally inherited, thereby creating a society that is largely female-kin biased^20,68,69^. While our results support this at the level of lack of agonism dominance, this was not supported in the agonistic competition data. Further, Xia *et al*.^70,71^ demonstrated a tendency for Tibetan macaque males and females to most frequently groom up the hierarchy and with those of adjacent rank; however, we found no evidence to support this claim across either lack of agonism or agonistic competition ranks. Again, this could be due to a lack of dominance-related data and the proportions of unknown relationships as well as possible differences in dominance rank analyses.

The marked direction of the difference in linearity or steepness (*h′*) of the chimpanzee (>0.90) and Tibetan macaque groups (<0.35) is not intuitive given the differences in dominance style between chimpanzees (relaxed/egalitarian, less steep) and Tibetan macaques (grade-two despotic, steeper). However, linearity scores are largely inconsistent and significantly reduced in matrices with >50% unknown relationships^7,77–79^; therefore, we attributed this inconsistency in linearity scores of the Tibetan macaques to the large proportion of unknown relationships (null dyads) in matrices (Supplementary Tables S5–S6). Such investigations of unknown relationships on Elo-rating’s stability metric are unknown; however, the temporal interactions diagrams from the Elo-ratings for the Tibetan macaque group (Supplementary Figs S13–S14) show few (if any) rank reversals across this limited study period and provide evidence to discount the linearity scores and support the high calculations of the Elo-rating procedure’s stability value (0.99, Table 3). Therefore, we consider the Elo-rating stability metric most appropriate to depict the high consistency of dominance interactions in the Tibetan macaque group. However, it is also possible that the large disparity in the amount of data collected and number of individuals observed between the two populations account for unlikely comparisons. While this disparity is not preferred, many primatological investigations use all-occurrence sampling of dominance interactions to derive ranks as performed here. We suggest that comparable, yet species-specific, data collection methods and statistical analyses between authors and investigations are necessary to maximize the collection of dominance interactions and generate commensurate results.

Drews^21^ proposes that (1) the functional appeal of calculating dominance hierarchies is to predict future interactions between individuals across multiple measures of sociality, and (2) that this predictive value is one of the only ecologically valid reasons to use dominance analyses. However, few of our derived ranks across dominance contexts correlated with patterns of other social behaviors in either the chimpanzee or Tibetan macaque group. Regardless, our results allow for continued discussion of the basis of dominance, both structurally and functionally.

Flack and de Waal^19^ posit that social power and dominance status are best measured through formal signals of subordination; our results generally support this claim. Across both species, dominance contexts that better represent formal, perceived, or agreed-upon dominance status (i.e., lack of agonism, privileged role, and access to resources) resulted in greater cross-context predictability than ranking structures that were derived from agonistic competitions and aggressiveness (Tables 3, 7, 8). Specifically, submissions and fleeing/yielding upon aggression constituted lack of agonism dominance in both chimpanzee and Tibetan macaque groups, directional grooming by the chimpanzees constituted privileged role dominance, and order when entering newly accessible enclosure spaces with food resources constituted access to resources dominance. These three measures represent metrics that are largely comprised of formal signals of subordination (or independently unchallenged orderings) and were found to correlate (at differing degrees) with networks of other social behaviors. Therefore, we suggest that future investigations of dominance in nonhuman primates should shift emphasis from purely agonistic interactions to multiple behavioral contexts that can lend to larger conversations of individual roles, structural attributes, differences in status and trends of signaling formal subordination.

Before exploring the effect of dominance on other systems (e.g., biological, behavioral, or reproductive), we suggest future investigations (1) construct hierarchies from various species-relevant contexts and (2) validate their model(s) to justify its appropriateness. While our results found high within-context ranking reliability, this does not indicate that such an exhaustive list is necessary in other investigations. However, the non-linear assumptions of ADAGIO and predictive probabilities of Elo-rating make them favorable approaches by these authors. In this investigation, we propose a sequence of analyses to validate such models across contexts and analyses that could be implemented by other investigators.

Our approach to juxtapose dominance rank analyses and derive various context-dependent dominance structures contributes to larger conversations on dominance style, correlates with other behavioral methods, methodological considerations, and statistical inconsistencies. With current and past variability in behavioral measures of dominance and statistical methods to derive dominance rankings, it is imperative to test methodologies and comparative configurations of computational analyses against ecological validity and theoretical soundness. We were able to cross-check such analyses by using data from two primate species, in four different dominance contexts, analyzed with five different dominance ranking statistics, and compared across five other social networks. These results do not just speak to the structures of dominance and their social correlates in these two primate groups but also contribute to broader considerations of how to define, measure, test, and validate dominance as a construct. Specifically, our results indicate the presence of context-dependent dominance and individual social roles in the captive chimpanzee group, one broadly defined dominance structure in the Tibetan macaque group, high within-rank reliability, but little cross-context predictability, as well as supported notions of formalized signals of subordination as the most insightful measures of dominance and unknown relationships (null dyads) having notable impact on these analyses.

Overall, we suggest that this comparative approach is preferred over more narrowly defined investigations of dominance where one or few behavioral metrics and statistical analyses are considered with little further investigation of rank reliability or cross-context predictability. We urge the use of similar techniques in future investigations of dominance across other fields, specifically, this comparative approach might be of interest to those investigating nonhuman primates^80^, cetaceans^80,81^, elephants^82,83^, wolves^84^, dogs^85^, or other species living in complex social groups^86^.

## Methods

### Study Sites and Individuals

#### Captive chimpanzees

J.A.F observed one group of captive chimpanzees (*Pan troglodytes*) at Chimpanzee Sanctuary Northwest (CSNW) in Cle Elum, WA. This chimpanzee group was composed of one male and six females (*N* = 7), ages 34 to 44 years (40.7 ± 3.1) with no known genetic relatedness (Table 2). Little is known about each individual’s specific early life history; however, all were from pet homes, the entertainment industry, wild-caught, or laboratory-born. All seven chimpanzees retired to CSNW from a biomedical research facility in June 2008. The seven chimpanzees have been exclusively housed together since arriving at CSNW. The chimpanzees have systematic access to a total of seven conjoined enclosure spaces: three small indoor rooms (~9.5 m^2^ each), one slightly larger indoor room (~13 m^2^), one large two-story indoor room (~111 m^2^), one indoor-outdoor space (caged walls with solid roof and bark substrate) with climbing structures (~56 m^2^), and one large open-topped outdoor space (electric-fenced, earth substrate) with multiple climbing structures (~1 ha). The chimpanzees were provided with three meals (either individually served or forage-style) and one to two small forages (to motivate shifts in enclosure access) per day, water *ad libitum*, various environmental enrichment throughout each morning and food-puzzle enrichment each evening.

#### Tibetan macaques

J.A.M and L.K.S observed one group (Yulingkeng A1) of wild Tibetan macaques (*Macaca thibetana*) at the Valley of the Wild Monkeys in the Huangshan Scenic District, Anhui Province, China^87^. For the current investigation, we only used data from adult-adult interactions, where an adult was above the age of six years at the time of data collection. Therefore, we focused our investigation on 24 adults: 13 females and 11 males between the ages of 6 and 31 years (M = 14.86 ± 7.63) (Table 2). The Yulingkeng A1 group has been habituated to human research by Anhui University since 1986 and human tourism since 1994^88,89^. The monkeys are provisioned with corn three to four times per day by park staff in the presence of tourist and researchers^87^. The YA1 group is free-ranging (but managed by park staff) across the park’s provisioning zone, manufactured tourist/researcher platforms and bridges, a stream and waterfall, forests, and cliffs.

### Behavioral Data Collection

#### Captive chimpanzees

J.A.F collected behavioral data on three randomly assigned days per week from June 15 to August 28, 2017 (N = 31 days) from 9:00 to 13:00 using a combination of all-occurrence and instantaneous scan sampling^90^. For both sampling methods, we utilized the same ethogram to operationally define and categorize behaviors that were derived specifically for this investigation or have been modified from the American Zoological Association^91^ and Jane Goodall Institute^92^ (see Table 1). J.A.F collected behavioral data on an iPad (2nd generation) using Zoo Monitor^93^.

We employed all-occurrence data collection during all observable social interactions (aggressive and/or affiliative) and shifts in access between enclosure spaces as the chimpanzees gained access to forage meals. We did not observe a single all-occurrence interaction for longer than 15 minutes (typically, bouts of allogrooming). We recorded social interactions as single events (occurrences) of unidirectional interaction with static membership; therefore, changes in individual involvement or direction constituted a separate event. We collected instantaneous scan samples between all-occurrence observations at no less than 15-minute intervals. During scan sampling, we observed each chimpanzee individually in sequence as encountered during sweeps from south to north across the facility; when individuals were on the same longitudinal line, they were observed from west to east. During data collection, we prioritized the collection of all-occurrence data (social behaviors) over scan data (nearest neighbor associations). If all-occurrence social interactions were observed during scan sampling, partially completed scans were discarded or quickly completed if all the remaining chimpanzees could be seen from that current location. Because the timing of shifts in-between enclosure spaces were decided by CSNW staff, scan samples were not conducted if these events were about to occur; however, if shifts in access were initiated by caregivers during scan sampling, the interrupted scan was discarded. This research complied with the protocol issued to J.A.F approved by Central Washington University’s Institutional Animal Care and Use Committee (protocol: A041701) and all methods performed are in accordance with relevant guidelines and regulations. All data generated or analyzed during this study are included in this published article (Supplementary Tables S1–S4).

#### Tibetan macaques

L.K.S collected behavioral data from July 14 to August 27, 2016 (N = 36 days) from 7:00–12:00 and 14:00–17:00 daily. L.K.S utilized all-occurrence sampling to collect agonistic data^90^. Agonistic data consisted of fear-grin, scream, flee, displace, threat, lunge, chase, grab, slap, and bite as defined by Berman *et al*.^30^. J.A.M collected behavioral data from July 12 to August 8, 2016 (N = 25 days) from 7:00–12:00 and 14:00–17:00 daily. J.A.M utilized 10-minute focal-animal sampling focused on adult females to collect allogrooming data^90^. Individual maternal kinship relations are well documented by researchers at Anhui University. We calculated maternal kinship relationships on an eight-point scale (0 = no known genetic relatedness, 8 = twin siblings) with missing values (empty cells) for immigrant males and other unknown relationships. The research outlined here adhered to the American Society of Primatologists (ASP) Principles for the Ethical Treatment of Non-Human Primates. This research also complied with the protocols approved by Central Washington University’s Institutional Animal Care and Use Committee (protocols: A041606 and A051602), and all methods performed are in accordance with relevant guidelines and regulations. All data generated or analyzed during this study are included in this published article (Supplementary Tables S5–S6).

### Statistical Analyses

Following conventional methods of analyzing dominance hierarchies within primate populations, we coded unambiguously-directed *agonistic interactions* (or “competitions”) in a 1:0 dichotomous fashion, where 1 indicated the “actor” who “won” the interaction, and 0 indicated the “recipient” who “lost” the interaction. For these reasons, submissive behaviors (*lack of agonism*) were reverse-coded, where the actor was said to have lost (0) to the winning (1) recipient. Chimpanzees who accessed newly opened enclosure spaces (*access to desired resources*) were coded to “win” against all the “losing” chimpanzees who entered later (i.e., access order = A, B, C; A “beat” B, A “beat” C, B “beat” C). *Privileged role* interactions were measured through directional occurrences of allogrooming in the captive chimpanzee group where the grooming recipient (the groomee) was coded as the “winner” and the bout’s actor (the groomer) was coded to “lose” the interaction. For grooming bouts between more than two individuals (polyadic grooming), bouts were coded in a directional dyadic fashion.

#### Dominance hierarchy

For each dominance context (see Table 1), we derived dominance ranks for each individual in both groups using the following analyses:

*David’s scores* (hereafter, DS) are the most conventional and derive a dominance index for each individual so that those typically “dominating” have a large positive score, and those that are typically “dominated” have large negative scores. David’s scores calculate the proportion of wins over losses in agonistic competitions relative to the total number of observed interactions and are corrected for chance occurrences of observed outcomes^46^.

The *I & SI method* (hereafter, I&SI) aims to maximize rank orders most consistent with linear structures, thereby minimizing the number of individual ranking inconsistencies (or rank reversals) and the statistical power of such inconsistencies^94^. To test for hierarchical ranking order linearity, many researchers conduct de Vries’^47^ test for linearity (*h*′)^10,39,41^. This test (*h*′) derives the certainty (or steepness; 0.00–1.00) of dominant individuals always acting (or “winning”) in interactions over a recipient (“loser” or subordinate)^47,95,96^.

Neumann *et al*.^24^ proposed using a non-matrix-based sequential analysis of dominance: *Elo-ratings*. Elo-ratings utilize the observed outcomes of dominance interactions to calculate the individual probability of success against all other individuals in the future to create a temporal and sequential analysis of dominance rankings. Using predictive probability based on previously observed interactions, Elo-ratings employ a “winner benefit” and “loser tax” paradigm, where the more an individual “wins” interactions, the more likely they are to win future interactions^97^. This statistical package also quantifiably characterizes *dominance stability*, as measured through the stability of each individual’s Elo rankings over time. Specifically, this stability characteristic calculates the ratio of rank changes per individual over a given period of time, with large variation in individuals’ ranks across the entire population resulting in low stability (score closer to 0.00) and no/small variations in individuals’ ranks resulting in high stability (score closer to 1.00)^24^. Elo-rating procedures also calculate the *percent of dyads* that were not observed to interact at all over the study period; this metric is known as the percent of null dyads.

Fushing, *et al*.^31^ devised *PERC* to investigate dominance relationships that may be nonlinear in nature. By inferring the rank potential for all individuals, minimizing errors, and computing confidence bounds for selected features, these authors proposed a novel and complex ranking system. PERC utilizes non-linear methods to derive relative ranks with individual confidence ratings (represented via heat maps); confidence ratings near chance (50%) indicate shared or inconsistent ranks of individuals^31^.

Recently, Douglas *et al*.^6^ developed *ADAGIO* (approach for dominance assessment in gregarious species) to analyze dominance without an underlying assumption of linearity and created a statistical package for testing both linear and nonlinear systems^98^, including linear, triangular, pyramidal, or a system of classes. ADAGIO extracts directed acyclic graphs from a given set of dyadic interactions to derive dominance structures free of the underlying assumptions of the structure’s organization^6^.

To investigate the degree of linearity within the groups’ dominance hierarchies, we calculated de Vries’ *h*′ test for interaction linearity (or certainty)^47^ for all dominance contexts in SOCPROG with 1000 permutations^95,96^. To accompany this linearity metric, we also calculated dominance stability and percent of null dyads (dyads without observed interactions) using Elo-rating procedures in R^24,99^.

#### Dominance rank reliability

To test for reliability of individual rank assignments across all statistical analyses (both within and between dominance contexts), we calculated Spearman’s *rho* correlation coefficients in R^99^. We used these analyses to examine if the various dominance statistics calculated similar ranks for each individual within the *same* dominance context. Few significant correlations would indicate a lack of reliability between these different analyses and emphasize the need for caution in selecting and interpreting dominance rank results for a given population. Further, to test for ranking correlations *between* dominance contexts, we also calculated each individual’s median rank across all ranking procedures (within each context) and compared these median ranks across contexts using Spearman’s *rho* correlation coefficients in R^99,100^.

#### Behavioral cross-context predictability

To test for the ecological validity of dominance and the ability of dominance ranks to predict patterns of other social behaviors, we calculated each individual’s median rank within each dominance context. We then converted these lists of median ranks into rank difference matrices (median rank of A - median rank of B) to investigate relationships between differences in dominance statuses and trends in the directionality of behaviors in other social contexts using QAP multiple matrix regression analyses in UCINET^73^. For the captive chimpanzee data, we investigated the relationships between all four dominance contexts and networks of all affiliation, agonism, and nearest-neighbor associations (see Table 1). For the free-ranging adult Tibetan macaque data, we investigated the relationships between both dominance contexts and occurrences of grooming and maternal relatedness (kinship) (see Table 1). The dominance rank(s) with the most predictability (significant correlations with other social measures) may provide the best insight into the ranking pattern with the most ecological validity and highest predictive value^21^.

## Electronic supplementary material

Supplementary Figures S1-S18

Supplementary Tables S1-S6

## References

[1] Alvarez, F. Social hierarchy under different criteria in groups of squirrel monkeys, *Saimiri sciureus*. *Primates* **16**, 437–455 10.1007/BF02382741(1975).

[2] Bernstein IS (1981). Dominance: The baby and the bathwater. J. Behav. Brain Sci..

[3] de Waal, F. B. M. Dominance “style” and primate social orgnaization. *Comparative* Socioecology (ed. Sanden, V. F.) 243–264 (Blackwell Scientific, 1989).

[4] Richards SM (1974). The concept of dominance and methods of assessment. Anim. Behav..

[5] Bang A, Deshpande S, Sumana A, Gadagkar R (2010). Choosing an appropriate index to construct dominance hierarchies in animal societies: A comparison of three indices. Anim. Behav..

[6] Douglas PH, Ngonga Ngomo AC, Hohmann G (2017). A novel approach for dominance assessment in gregarious species: ADAGIO. Anim. Behav..

[7] Klass K, Cords M (2011). Effect of unknown relationships on linearity, steepness and rank ordering of dominance hierarchies: Simulation studies based on data from wild monkeys. Behav. Processes.

[8] Norscia I, Palagi E (2015). The socio-matrix reloaded: from hierarchy to dominance profile in wild lemurs. PeerJ.

[9] Sánchez-Tójar A, Schroeder J, Farine DR (2017). A practical guide for inferring reliable dominance hierarchies and estimating their uncertainty. J. Anim. Ecol..

[10] Vervaecke H, De Vries H, Van Elsacker L (2000). Dominance and its behavioral measures in a captive group of bonobos (*Pan paniscus*). Int. J. Primatol..

[11] Thierry B (1990). Feedback loop between kinship and dominance: The macaque model. J. Theor. Biol..

[12] Sapolsky RM (2005). The influence of social hierarchy on primate health. Science.

[13] Cowlishaw G, Dunbar RI (1991). Dominance rank and mating success in male primates. Anim. Behav..

[14] Foster MW (2009). Alpha male chimpanzee grooming patterns: Implications for dominance “style. Am. J. Primatol..

[15] Seyfarth RM (1977). A model of social grooming among adult female monkeys. J. Theor. Biol..

[16] Murray CM, Eberly LE, Pusey AE (2006). Foraging strategies as a function of season and rank among wild female chimpanzees (*Pan troglodytes*). Behav. Ecol..

[17] Murray CM, Mane SV, Pusey AE (2007). Dominance rank influences female space use in wild chimpanzees, Pan troglodytes: towards an ideal despotic distribution. Anim. Behav..

[18] Wittig RM, Boesch C (2003). Food competition and linear dominance hierarchy among female chimpanzees of the Tai National Park. Int. J. Primatol..

[19] Flack, J. C. & de Waal, F. B. M. Dominance style, social power, and conflict management. *Macaque societies: A model for the study of social organization* (eds Theirry, B., Singh, M., & Kaumanns, W.) 157–185 (Cambridge University Press, 2004).

[20] Thierry, B. Covariation of conflict management pattern across macaque species. *Natural Conflict Resolution* (eds Aureli, F., & de Waal, F. B. M.) 106–128 (University of California Press, 2000) (2000).

[21] Drews C (1993). The concept and definition of dominance in animal behavior. Behavior.

[22] Schjelderupp-Ebbe T (1922). Beitrage zur sozialpsychologie des haushuhns. Zeitschnift Für Psychoanalytische Pädagogik.

[23] Candland DK, Hoer JB (1981). The logical status of dominance. Behav. Brain Sci..

[24] Neumann C (2011). Assessing dominance hierarchies: Validation and advantages of progressive evaluation with Elo-rating. Anim. Behav..

[25] Hinde, R. A. & Stevenson-Hinde, J. Towards understanding relationships: Dyadic stability. *Growing Points in Ethology* (eds Bateson, P. P. G. & Hinde, R. A.) 451–479 (Cambridge University Press, 1976).

[26] Bayly KL, Evans CS, Taylor A (2006). Measuring social structure: A comparison of eight dominance indices. Behav. Processes.

[27] Noė R, de Waal FBM, van Hooff JA (1980). Types of dominance in a chimpanzee colony. Folia Primatol..

[28] Paoli T, Palagi E, Tarli SMB (2006). Reevaluation of dominance hierarchy in bonobos (*Pan paniscus*). Am. J. Phys. Anthropol..

[29] Hinde, R. A. *Biological bases of human socal behaviour* (McGraw-Hill, 1974).

[30] Berman CM, Ionica CS, Li J (2004). Dominance style among *Macaca thibetana* on Mt. Huangshan, China. Int. J. Primatol..

[31] Fushing H, McAssey MP, Beisner B, McCowan B (2011). Ranking network of a captive rhesus macaque society: A sophisticated corporative kingdom. PLoS ONE.

[32] Kaburu SSK, Newton-Fisher NE (2015). Egalitarian despots: Hierarchy steepness, reciprocity and the grooming-trade model in wild chimpanzees, Pan troglodytes. Anim. Behav..

[33] Muller, M. N. Agonistic relations among Kanyawara chimpanzees. *Behavioural Diversity in Chimpanzees and* Bonobos (eds Boesch, C., Hohmann, G., & Marchant, L.) 112–123 (Cambridge University Press, 2002).

[34] Wakefield ML (2008). Grouping patterns and competition among female Pan troglodytes schweinfurthii at Ngogo, Kibale National Park, Uganda. Int. J. Primatol..

[35] Vessey SH (1981). Dominance as control. Behav. Brain Sci..

[36] Laporte MNC, Zuberbühler K (2010). Vocal greeting behaviour in wild chimpanzee females. Anim. Behav..

[37] Lu A, Koenig A, Borries C (2008). Formal submission, tolerance and socioecological models: A test with female Hanuman langurs. Anim. Behav..

[38] Ostner J, Heistermann M, Schülke O (2008). Dominance, aggression and physiological stress in wild male Assamese macaques (Macaca assamensis). Horm. Behav..

[39] Stevens JMG, Vervaecke H, De Vries H, Van Elsacker L (2007). Sex differences in the steepness of dominance hierarchies in captive bonobo groups. Int. J. Primatol..

[40] Clutton-Brock TH, Albon SD, Gibson RM, Guinness FE (1979). The logical stag: Adaptive aspects of fighting in red deer (*Cervus elaphus l*.). Anim. Behav..

[41] Leinfelder I, De Vries H, Deleu R, Nelissen M (2001). Rank and grooming reciprocity among females in a mixed-sex group of captive hamadryas baboons. Am. J. Primatol..

[42] Wilson, E. O. *Sociobiology: The new synthesis* (Harvard University Press, 1975).

[43] Arnold K, Whiten A (2003). Grooming interactions among the chimpanzees of the Budongo Forest, Uganda. Behavior.

[44] Fedurek P, Dunbar RIM (2009). What does mutual grooming tell us about why chimpanzees groom?. Ethology.

[45] Franz C (1999). Allowgrooming behavior and grooming site preference in captive bonobos (*Pan paniscus*): Association with female dominance. Int. J. Primatol..

[46] de Vries H, Stevens JMG, Vervaecke H (2006). Measuring and testing the steepness of dominance hierarchies. Anim. Behav..

[47] de Vries H (1995). An improved test of linearity in dominance hierarchies containing unknown or tied relationships. Anim. Behav..

[48] Mitani, J. C., Call, J., Kappeler, P. M., Palombit, R. A. & Silk, J. B. *The evolution of primate societies* (Chicago University Press, 2012).

[49] Boesch, C. & Boesch-Achermann, H. *The Chimpanzees of the Tai Forest: Behavioural Ecology and Evolution* (Oxford University Press, 2000).

[50] Stumpf, R. M. Chimpanzees and bonobos. *Primates in* Perspective (eds Campbell, C. J., Fuentes, A., MacKinnon, K. C., Bearder, S. K., & Stumpf, R. M.) 340–356 (Oxford University Press, 2011).

[51] Boesch C, Kohou G, Néné H, Vigilant L (2006). Male competition and paternity in wild chimpanzees of the Tai forest. Am. J. Phys. Anthropol..

[52] Goodall, J. *The Chimpanzees of Gombe: Patterns of Behavior* (Press of Harvard University Press, 1986).

[53] Muller MN, Wrangham RW (2004). Dominance, aggression and testosterone in wild chimpanzees: A test of the “challenge hypothesis.”. Anim. Behav..

[54] Pusey A (2008). Severe aggression among female Pan troglodytes schweinfurthii at Gombe National Park, Tanzania. Int. J. Primatol..

[55] Wakefield ML (2013). Social dynamics among females and their influence on social structure in an East African chimpanzee community. Anim. Behav..

[56] Funkhouser JA, Mayhew JA, Mulcahy JB (2018). Social network and dominance hierarchy analyses at Chimpanzee Sanctuary Northwest. PLoS ONE.

[57] Duncan LM, Jones MA, van Lierop M, Pillay N (2013). Chimpanzees use multiple strategies to limit aggression and stress during spatial density changes. Appl. Anim. Behav. Sci..

[58] Nieuwenhuijsen K, de Waal F (1982). Effects of spatial crowding on social behavior in a chimpanzee colony. Zoo Biol..

[59] Videan EN, Fritz J (2007). Effects of short- and long-term changes in spatial density on the social behavior of captive chimpanzees (*Pan troglodytes*). Appl. Anim. Behav. Sci..

[60] Kanngiesser P, Sueur C, Riedl K, Grossmann J, Call J (2011). Grooming network cohesion and the role of individuals in a captive chimpanzee group. Am. J. Primatol.

[61] Fraser ON, Schino G, Aureli F (2008). Components of relationship quality in chimpanzees. Ethology.

[62] Henzi SP, Barrett L (1999). The value of grooming to female primates. Primates.

[63] Shultz S, Dunbar R (2010). Bondedness and sociality. Behaviour.

[64] Silk J, Cheney D, Seyfarth R (2013). A practical guide to the study of social relationships. Evol. Anthropol..

[65] de Waal FBM (1986). The integration of dominance and social bonding in primates. Q.Rev. Biol..

[66] Newton-Fisher, N. E. & Kaburu, S. S. K. Grooming decisions under structural despotism: The impact of social rank and bystanders among wild male chimpanzees. *Anim. Behav*. **128**, 10.1016/j.anbehav.2017.04.012 (2017).

[67] de Waal FBM (1997). The chimpanzee’s service economy: Food for grooming. Ev. Hum. Behav..

[68] Zhao Q (1997). Intergroup interactions in Tibetan macaques at Mt. Emei, China. Am. J. Phys. Anthropol..

[69] Thierry, B. The macaques: A double-layered social organization. *Primates In Perspective* (eds Campbell, C. J., Fuentes, A., MacKinnon, K. C., Bearder, S. K., & Stumpf, R. M.) 229–241 (Oxford University Press, 2011).

[70] Xia D (2012). Grooming reciprocity in female tibetan macaques *Macaca thibetana*. Am. J. Primatol..

[71] Xia D (2013). Grooming reciprocity in male Tibetan macaques. Am. J. Primatol..

[72] IBM Corporation. *IBM SPSS Statistics for Windows* (IBM Corp., 2013).

[73] Borgatti, S. P., Everett, M. G. & Johnson, J. C. *Analyzing social networks* (Sage Publications, 2013).

[74] de Waal FBM, Luttrell LM (1985). The formal hierarchy of rhesus macaques: An investigation of the bared-teeth display. Am. J. Primatol..

[75] Merrick NJ (1977). Social grooming and play behavior of a captive group of chimpanzees. Primates.

[76] Watts DP (2006). Conflict resolution in chimpanzees and the valuable-relationships hypothesis. Int. J. Primatol..

[77] Hoy S, Bauer J (2005). Dominance relationships between sows dependent on the time interval between separation and reunion. Appl. Anim. Behav. Sci..

[78] Koenig, A. & Borries, C. The predictive power of socioecological models: A reconsideration of resource characteristics, agonism, and dominance hierarchies. *Feeding ecology in apes and other primates: Ecological, phsycical, and behavioral aspects* (eds Hohmann, G., Robbins, M. M., & Boesch, C.) 263–284 (Cambridge University Press, 2006).

[79] Vogel ER (2005). Rank differences in energy intake rates in white-faced capuchin monkeys, *Cebus capucinus*: The effects of contest competition. Behav. Ecol. Sociobiol.

[80] Yamagiwa, J. & Karczmarski, L. *Primates and Cetaceans: Field Research and Conservation of Complex Mammalian Societies* (Springer Science & Business Media, 2013).

[81] Radić, S, Connor, R. C., Sherwin, W. B. & Krützen, M. A novel mammalian social structure in Indo-Pacific bottlenose dolphins (*Tursiops* sp.): Complex male alliances in an open social network. *Proc. R. Soc. Lond. B. Biol. Sci*. **264**, 10.1098/rspb.2012.0264 (2012). 10.1098/rspb.2012.0264 PMC3385473 22456886

[82] Wittemyer G, Getz WM (2007). Hierarchical dominance structure and social organization in African elephants. Loxodonta africana. Anim. Behav..

[83] de Silva S, Ranjeewa AD, Kryazhimskiy S (2011). The dynamics of social networks among female Asian elephants. BMC Ecol..

[84] Cafazzo S, Lazzaroni M, Marshall-Pescini S (2016). Dominance relationships in a family pack of captive arctic wolves (*Canis lupus arctos*): The influence of competition for food, age and sex. PeerJ.

[85] Trisko RK, Smuts BB (2015). Dominance relationships in a group of domestic dogs (*Canis lupus familiaris*). Behaviour.

[86] Smith JE (2016). Leadership in mammalian societies: Emergence, distribution, power, and payoff. Trends Ecol. Evol..

[87] Berman CM, Li JH (2002). Impact of translocation, provisioning and range restriction on a group of *Macaca thibetana*. Int. J. Primatol..

[88] Berman CM, Li J, Ogawa H, Ionica C, Yin H (2007). Primate tourism, range restriction, and infant risk among *Macaca thibetana* at Mt. Huangshan, China. Int. J. Primatol..

[89] Li J, Wang Q, Han D (1996). Fission in a free-ranging Tibetan macaque troop at Huangshan Mountain, China. Chin. Sci. Bull..

[90] Altmann J (1977). Observational study of behavior: Sampling methods. Behaviour.

[91] Association of Zoos and Aquariums. Chimpanzee *(Pan troglodyte*s) Care Manual; aza.org/assets/2332/chimpanzeecaremanual2010r.pdf (2009).

[92] Jane Goodall Institute. ChimpanzeeZoo ethogram; ethosearch.org (1989).

[93] Ross, M. R. *et al*. *ZooMonitor* (2006).

[94] de Vries H (1998). Finding a dominance order most consitent with a linear hierarchy: a new procedure and review. Anim. Behav..

[95] Whitehead, H. *Analyzing Animal Societies: Quantitative Methods for Vertebrate Social**Analysis* (The University of Chicago Press, 2008).

[96] Whitehead H (2009). SOCPROG programs: Analysing animal social structures. Behav. Ecol. Sociobiol..

[97] Franz M, McLean E, Tung J, Altmann J, Alberts SC (2015). Self-organizing dominance hierarchies in a wild primate population. Proc. R. Soc. Lond. B. Biol. Sci..

[98] Preuschoft, S. & van Schaik, C. Dominance and communication. *Natural Conflict Resolution* (eds Aureli, F. & de Waal, F. B. M.) 77–105 (University of California Press, 2000).

[99] R Core Team. R: A language and enviornment for statistical computing (R Foundation for Stiatical Computing, 2016).

[100] de Silva S, Schmid V, Wittemyer G (2016). Fission–fusion processes weaken dominance networks of female Asian elephants in a productive habitat. Behav. Ecol..

