# Supplementary material for: Comparative Investigations of Social Context-Dependent Dominance in Captive Chimpanzees (Pan troglodytes) and Wild Tibetan Macaques (Macaca thibetana)
Source: Sci Rep. 2018 Sep 17;8:13909. doi: 10.1038/s41598-018-32243-2 (PMC6141571; doi:10.1038/s41598-018-32243-2)
Supplement: Supplementary file 1 — Supplementary Figures S1-S18 [file 41598_2018_32243_MOESM1_ESM.pdf]

Comparative Investigations of Social Context-Dependent Dominance in Captive Chimpanzees (*Pan troglodytes*) and Wild Tibetan Macaques (*Macaca thibetana*)

Jake A. Funkhouser[1*], Jessica A. Mayhew[1, 2], Lori K. Sheeran[1, 2], John B. Mulcahy[1, 2, 3], and Jinhua Li[4]

[1] Central Washington University, Primate Behavior Program, Ellensburg, 98926, USA
[2] Central Washington University, Department of Anthropology & Museum Studies, 98926, USA
[3] Chimpanzee Sanctuary Northwest, 98922, USA
[4] Anhui University, School of Resource & Environmental Engineering, 551, China
* jake.funkhouser@cwu.edu

Supplementary Figures

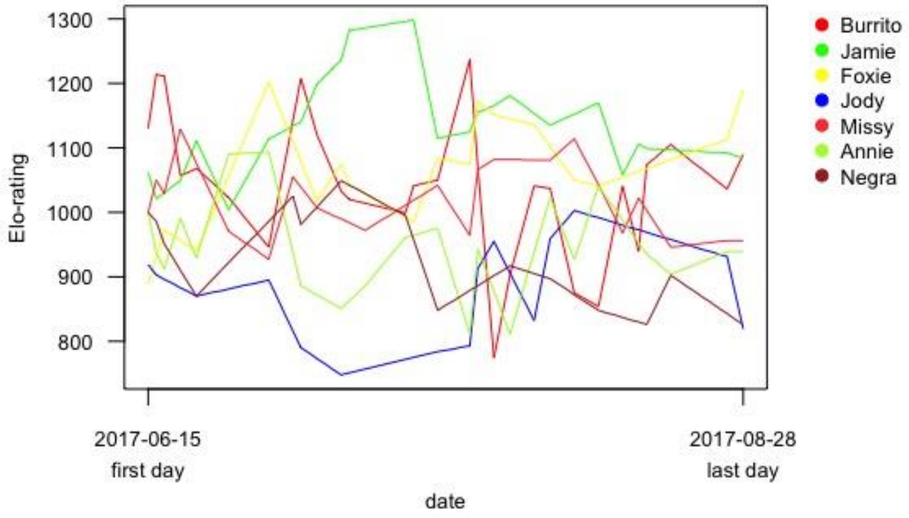

Supplementary Figure S1. Elo-rating diagram for chimpanzee agonistic competition

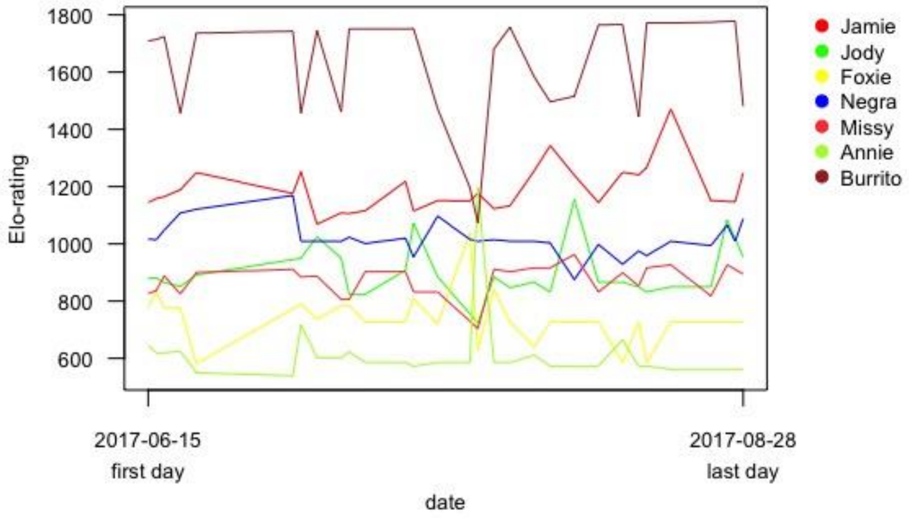

Supplementary Figure S2. Elo-rating diagram for chimpanzee lack of agonism

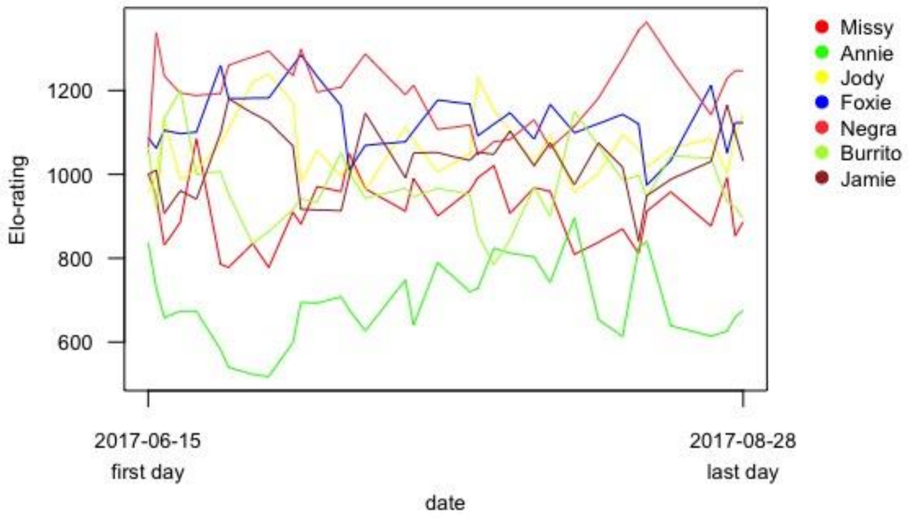

Supplementary Figure S3. Elo-rating diagram for chimpanzee privileged role

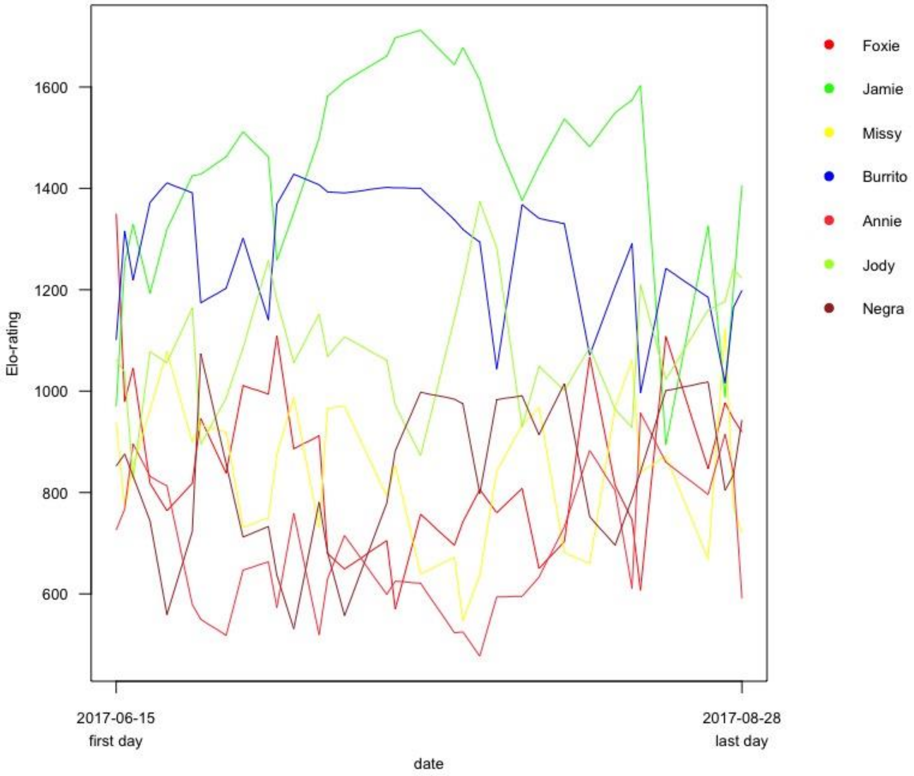

Supplementary Figure S4. Elo-rating diagram for chimpanzee access to resources

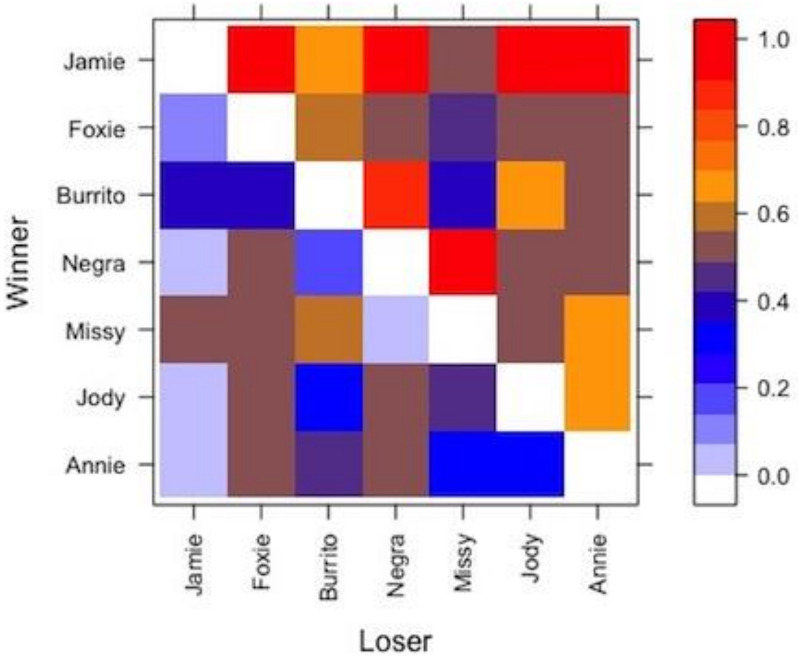

Supplementary Figure S5. PERC ranking certainty diagram for chimpanzee agonistic competition

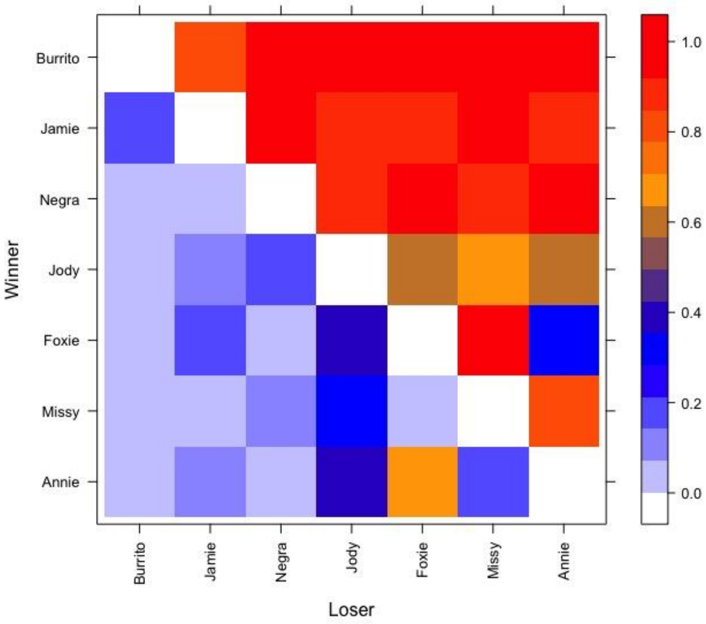

Supplementary Figure S6. PERC ranking certainty diagram for chimpanzee lack of agonism

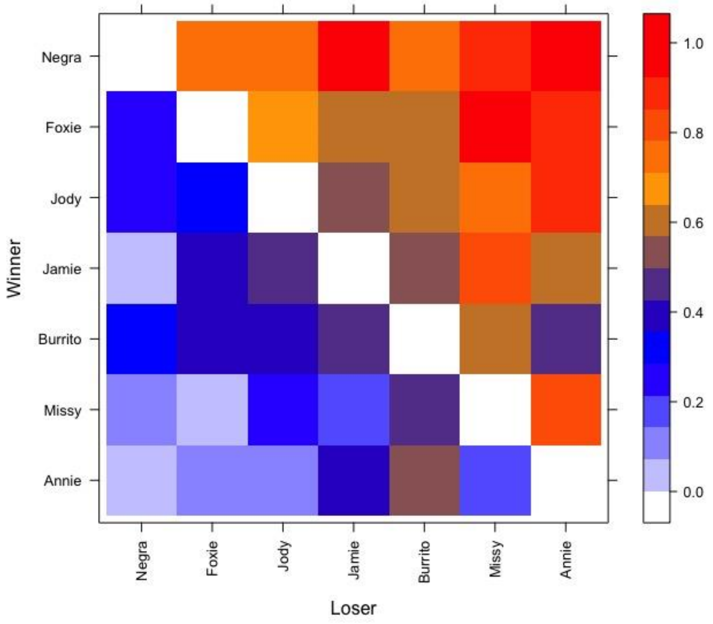

Supplementary Figure S7. PERC ranking certainty diagram for chimpanzee privileged role

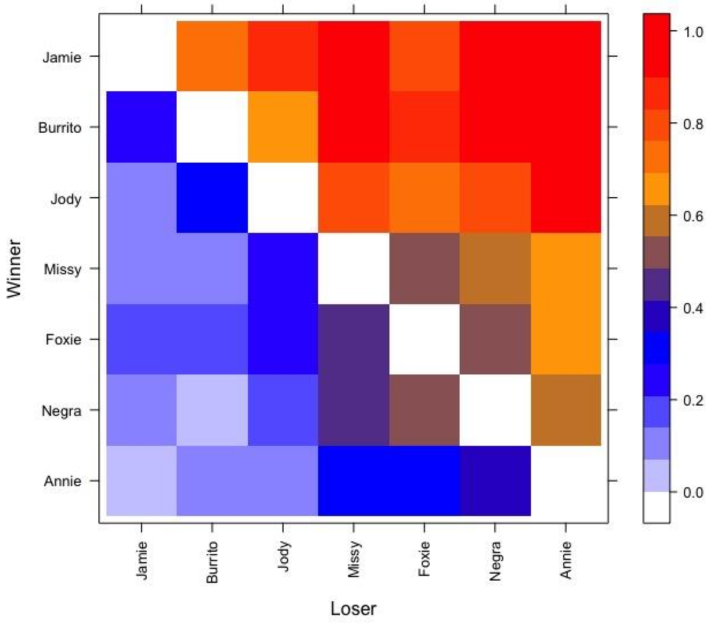

Supplementary Figure S8. PERC ranking certainty diagram for chimpanzee access to resources

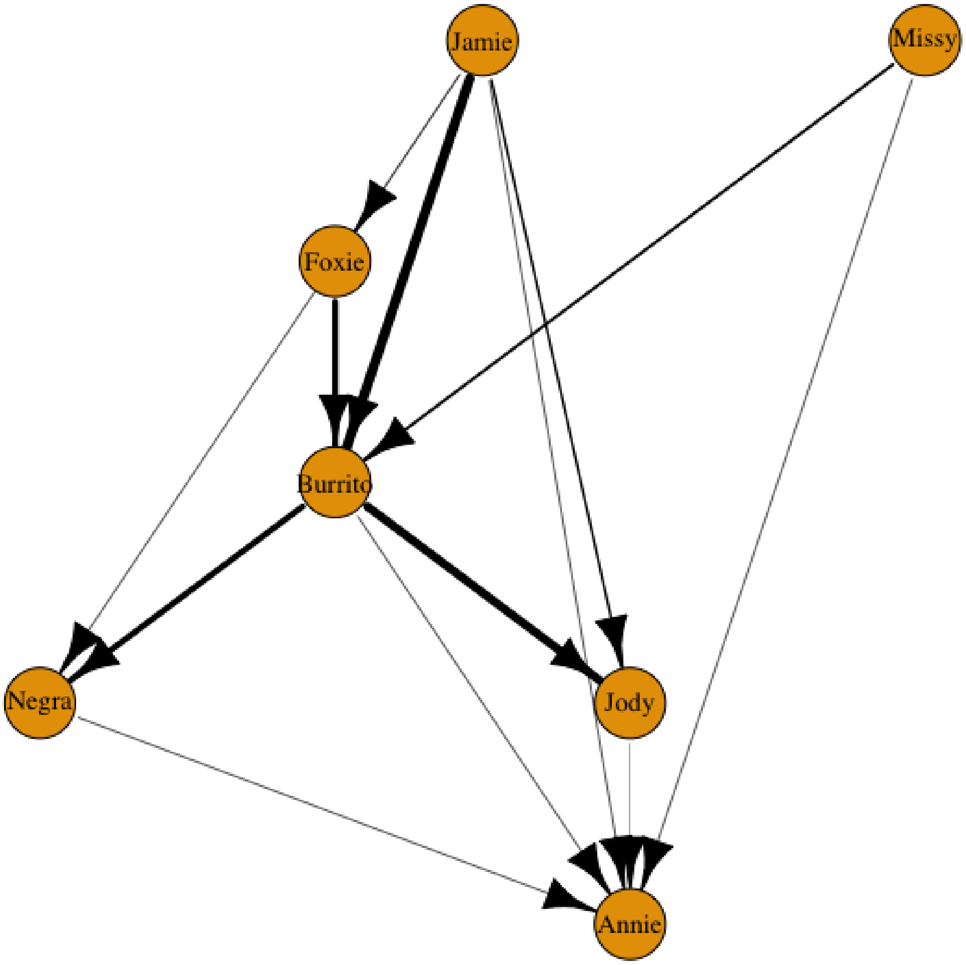

Supplementary Figure S9. ADAGIO diagram for chimpanzee agonistic competitions

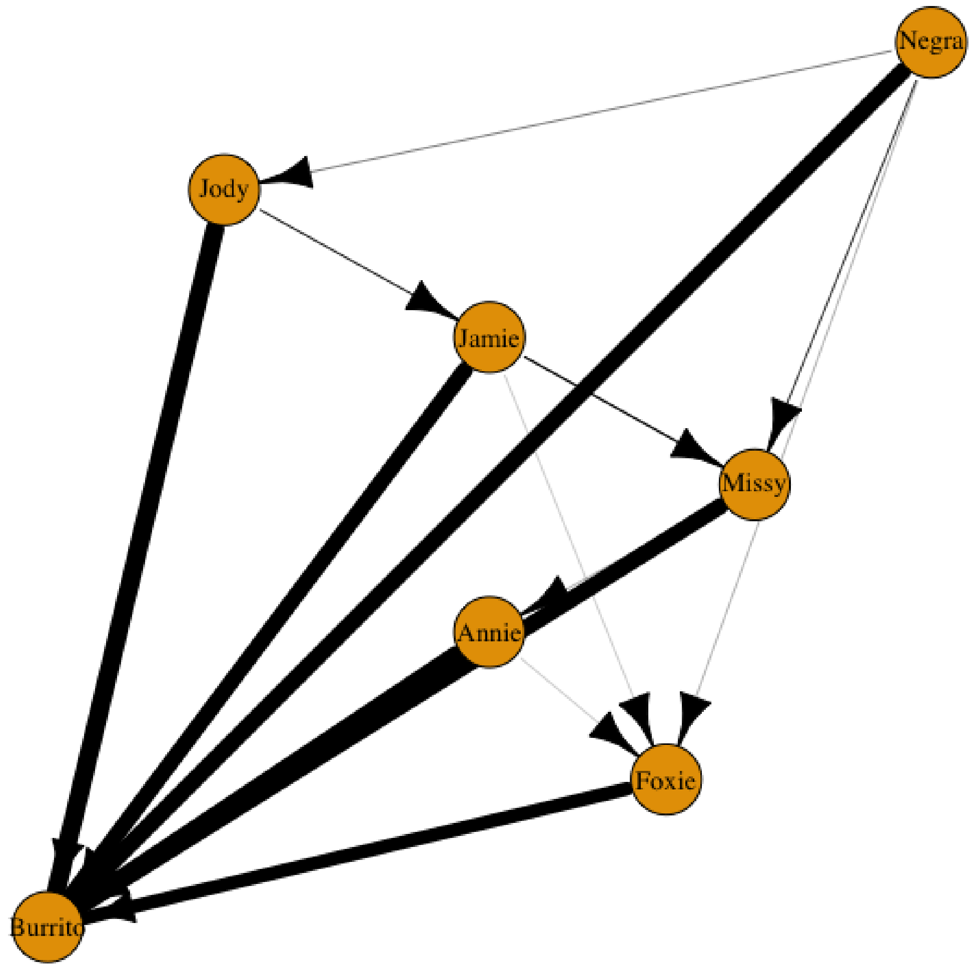

Supplementary Figure S10. ADAGIO diagram for chimpanzee lack of agonism

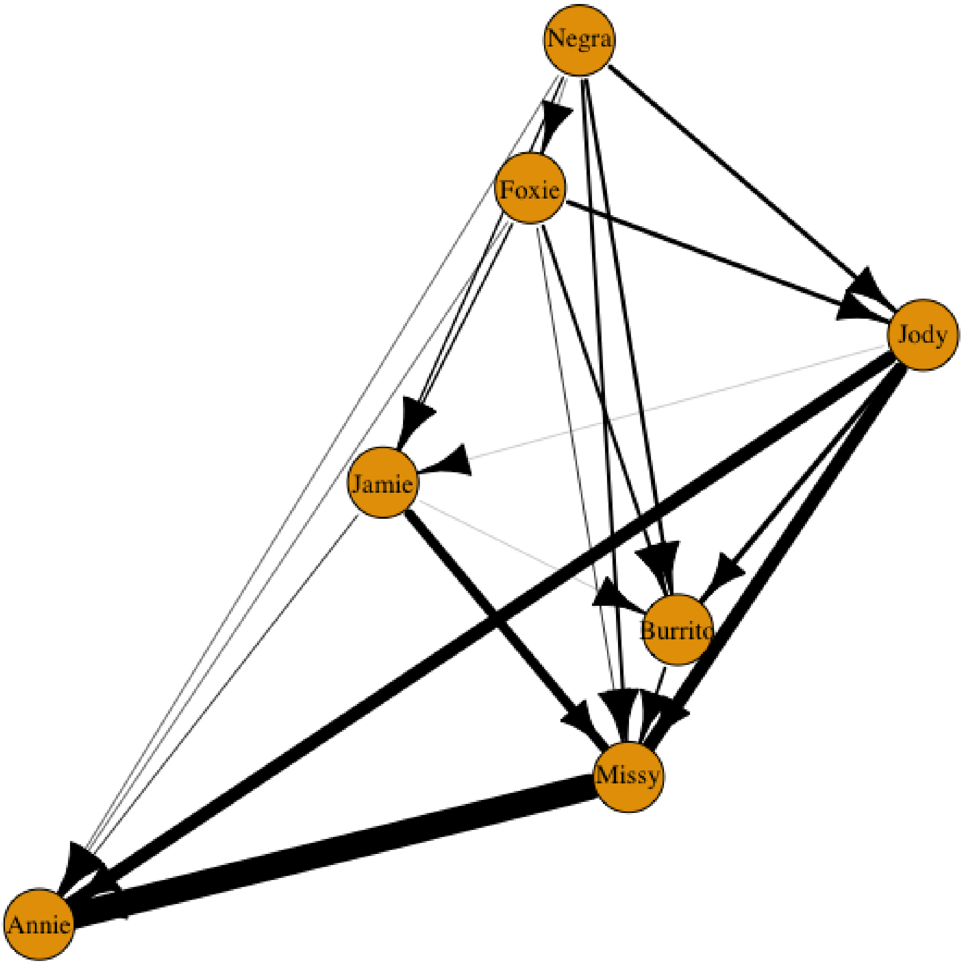

Supplementary Figure S11. ADAGIO diagram for chimpanzee privileged role

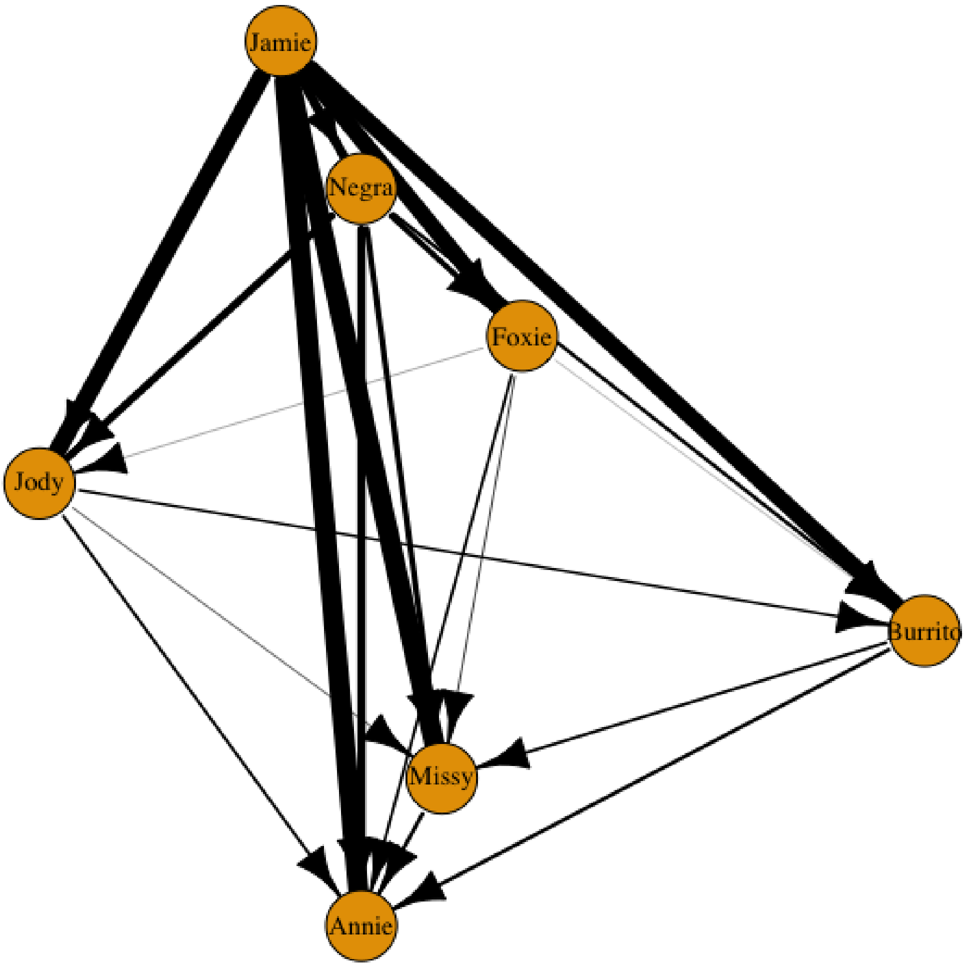

Supplementary Figure S12. ADAGIO diagram for chimpanzee access to resources

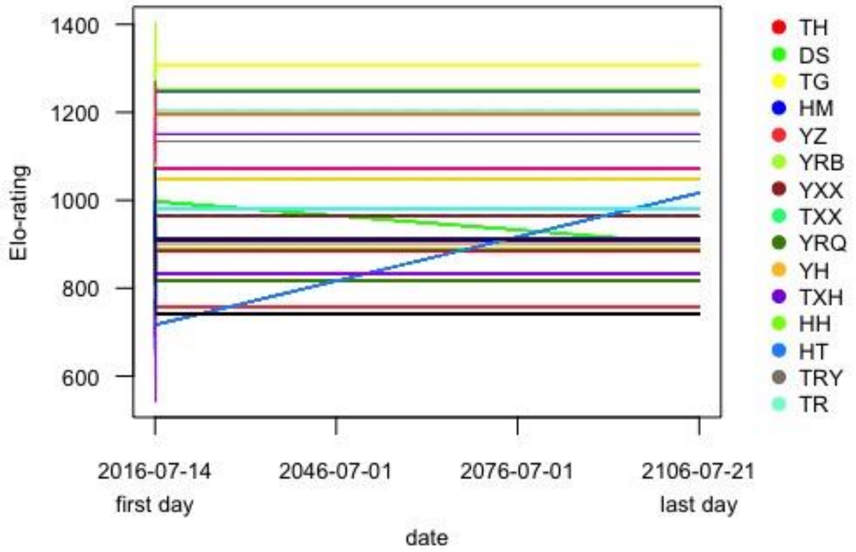

Supplementary Figure S13. Elo-rating diagram for Tibetan macaque agonistic competition

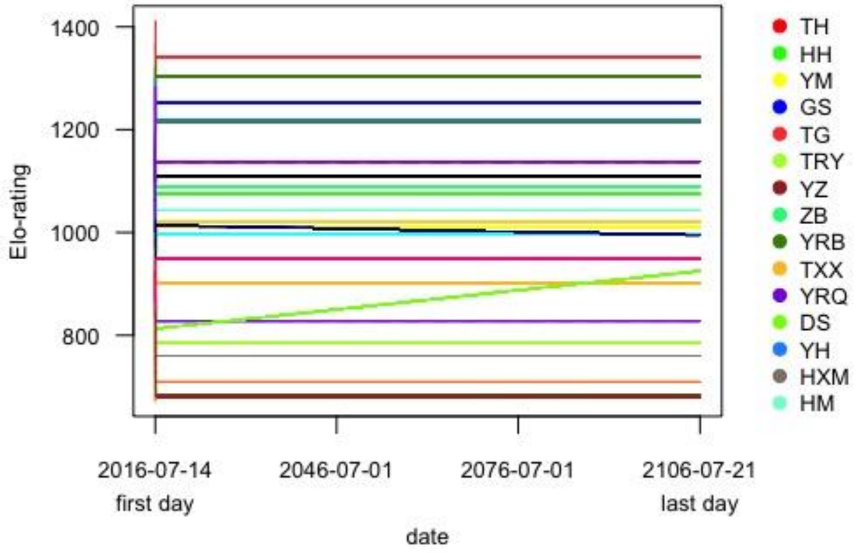

Supplementary Figure S14. Elo-rating diagram for Tibetan macaque lack of agonism

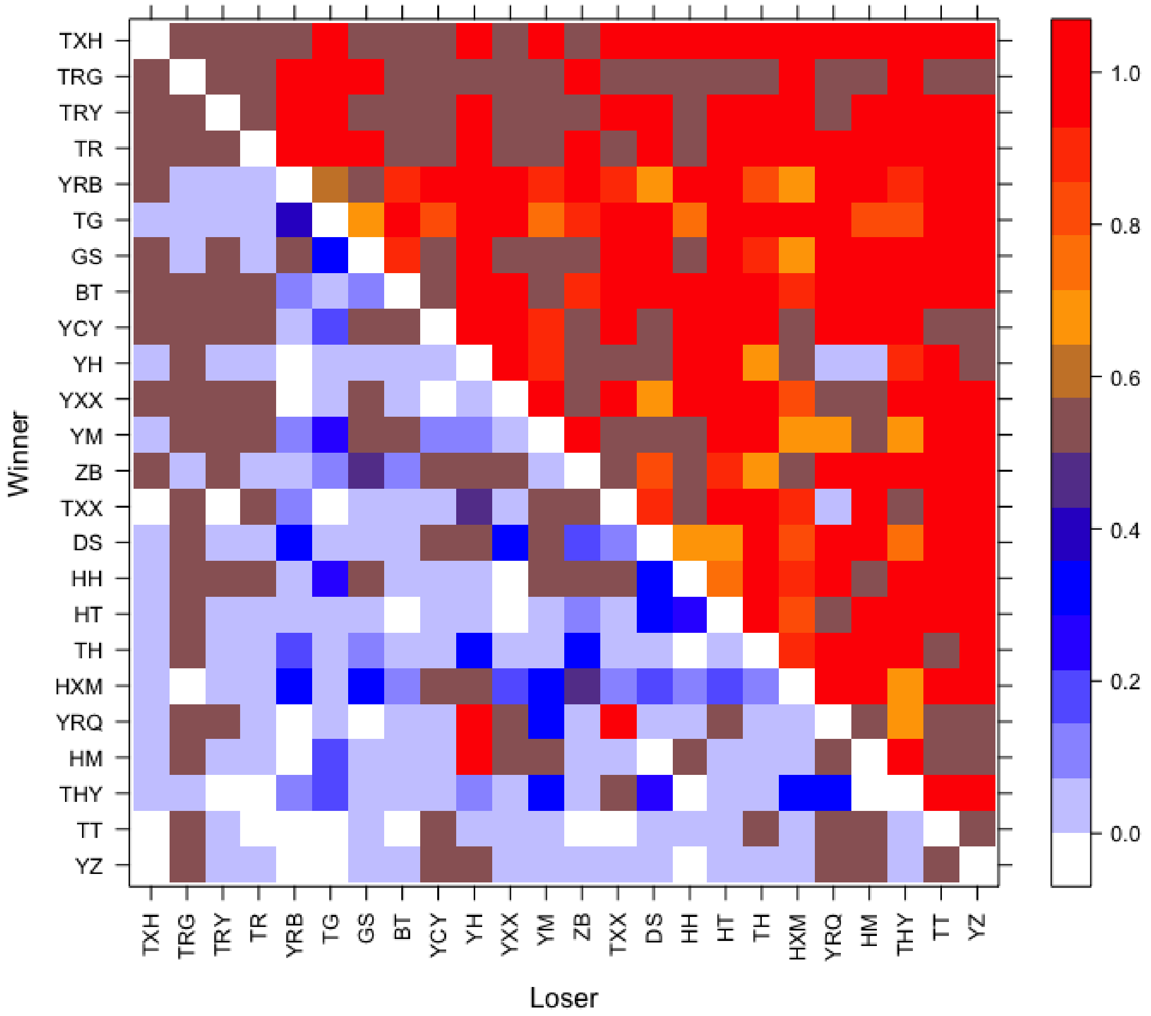

Supplementary Figure S15. PERC ranking certainty diagram for Tibetan macaque agonistic competition

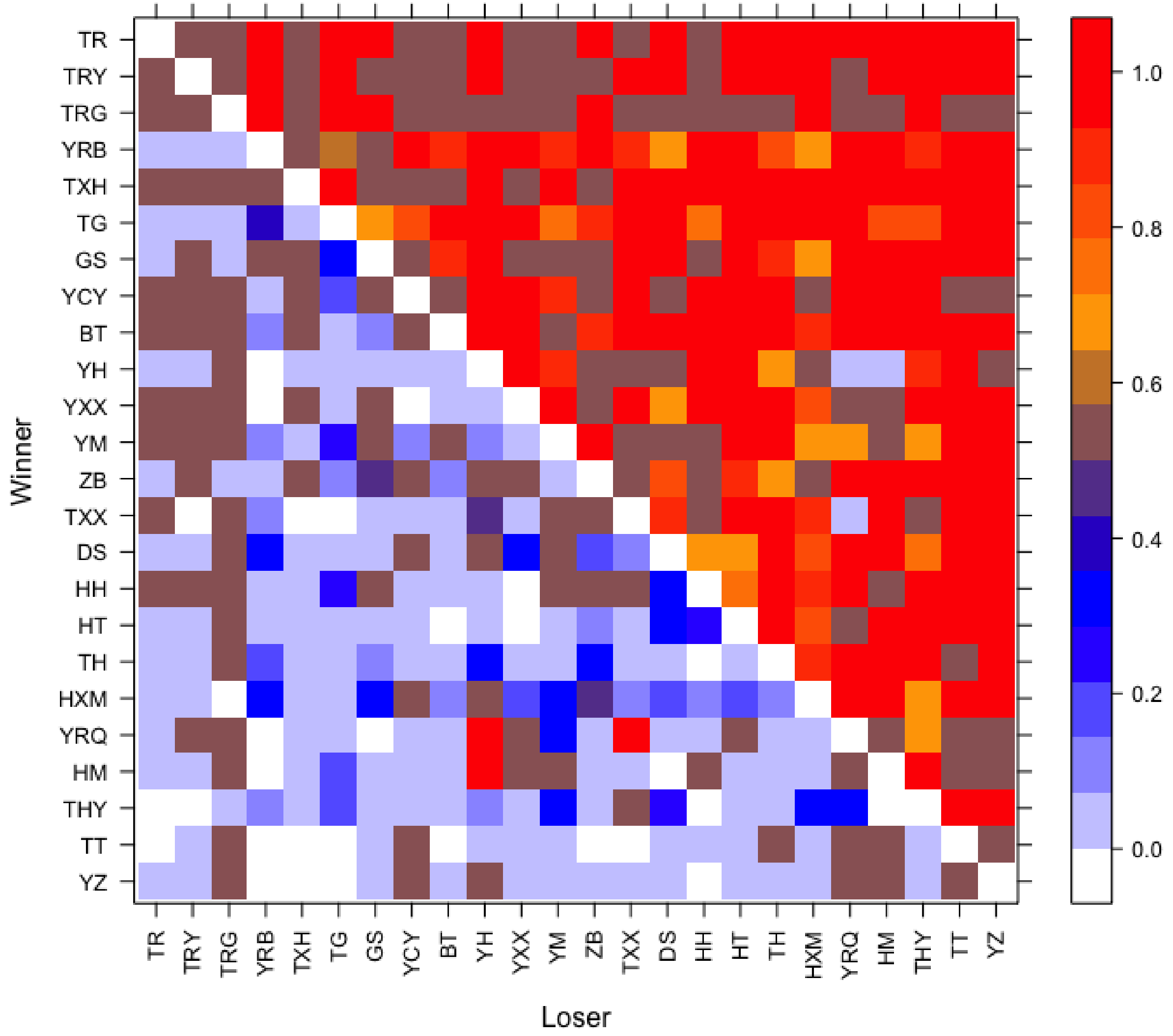

Supplementary Figure S16. PERC ranking certainty diagram for Tibetan macaque lack of agonism

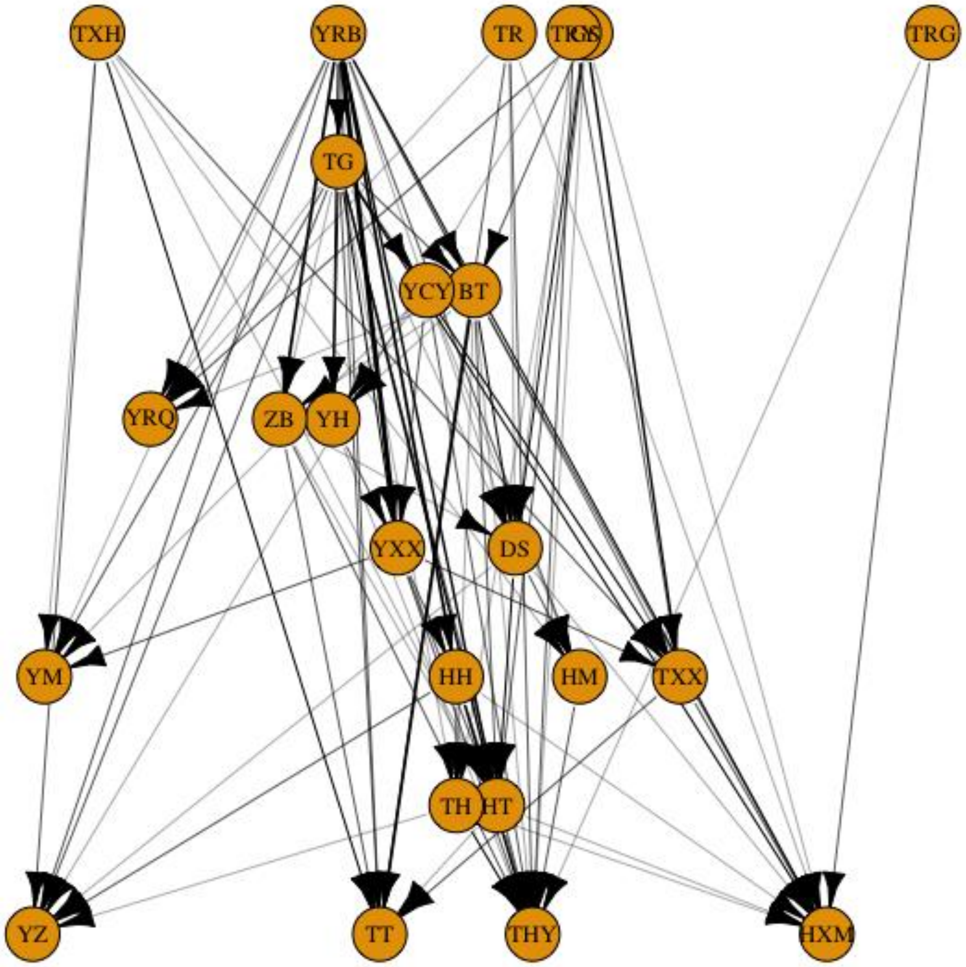

Supplementary Figure S17. ADAGIO diagram for Tibetan macaque agonistic competition

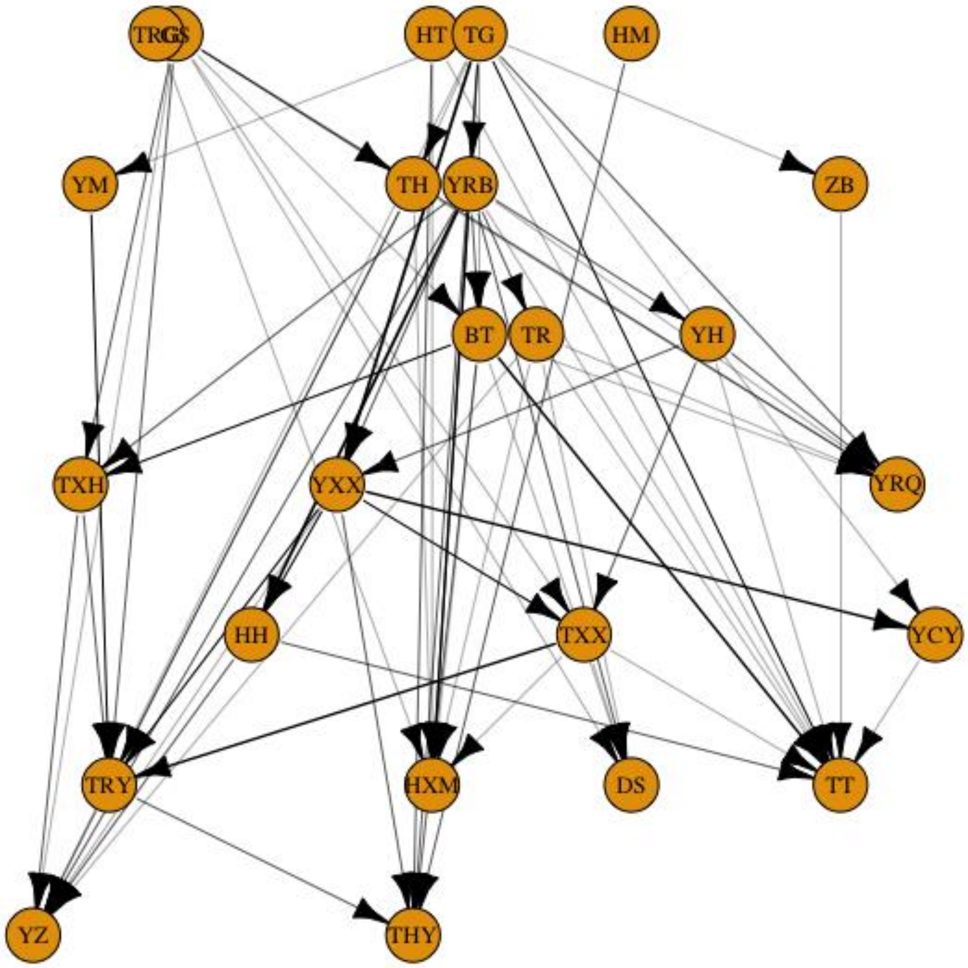

Supplementary Figure S18. ADAGIO diagram for Tibetan macaque lack of agonism
