# Supplementary material for: Comparative Investigations of Social Context-Dependent Dominance in Captive Chimpanzees (Pan troglodytes) and Wild Tibetan Macaques (Macaca thibetana)
Source: Sci Rep. 2018 Sep 17;8:13909. doi: 10.1038/s41598-018-32243-2 (PMC6141571; doi:10.1038/s41598-018-32243-2)
Supplement: Supplementary file 2 — Supplementary Tables S1-S6 [file 41598_2018_32243_MOESM2_ESM.pdf]

Comparative Investigations of Social Context-Dependent Dominance in Captive Chimpanzees (*Pan troglodytes*) and Wild Tibetan Macaques (*Macaca thibetana*)

Jake A. Funkhouser[1*], Jessica A. Mayhew[1, 2], Lori K. Sheeran[1, 2], John B. Mulcahy[1, 2, 3], and Jinhua Li[4]

[1] Central Washington University, Primate Behavior Program, Ellensburg, 98926, USA
[2] Central Washington University, Department of Anthropology & Museum Studies, 98926, USA
[3] Chimpanzee Sanctuary Northwest, 98922, USA
[4] Anhui University, School of Resource & Environmental Engineering, 551, China
* jake.funkhouser@cwu.edu

Supplementary Tables

	Annie	Burrito	Foxie	Jamie	Jody	Missy	Negra
Annie		23	0	0	1	2	2
Burrito	25		16	19	26	11	12
Foxie	0	26		0	0	0	0
Jamie	2	35	1		4	2	2
Jody	2	12	0	0		0	0
Missy	4	16	0	2	0		0
Negra	4	2	0	0	0	2	

Supplementary Table S1. Interaction matrix for chimpanzee agonistic competition data.

	Annie	Burrito	Foxie	Jamie	Jody	Missy	Negra
Annie		3	6	1	3	2	0
Burrito	105		82	86	122	104	96
Foxie	3	4		2	2	3	0
Jamie	9	21	12		14	18	6
Jody	5	4	3	2		2	1
Missy	14	1	0	1	1		1
Negra	1	2	3	0	6	11	

Supplementary Table S2. Interaction matrix for chimpanzee lack of agonism data.

	Annie	Burrito	Foxie	Jamie	Jody	Missy	Negra
Annie		14	1	11	14	33	0
Burrito	12		48	31	43	72	15
Foxie	7	68		36	52	8	3
Jamie	19	33	23		46	72	1
Jody	88	74	26	48		114	14
Missy	176	54	0	18	39		3
Negra	6	37	8	14	40	23	

Supplementary Table S3. Interaction matrix for chimpanzee privileged role data.

	Annie	Burrito	Foxie	Jamie	Jody	Missy	Negra
Annie		6	20	4	7	22	24
Burrito	66		55	18	49	66	63
Foxie	44	11		13	17	30	30
Jamie	67	55	53		64	66	59
Jody	65	25	49	9		57	53
Missy	49	7	35	6	16		36
Negra	39	2	30	6	12	28	

Supplementary Table S4. Interaction matrix for chimpanzee priority access to resources data.

	BT	DS	GS	HH	HM	HT	HXM	TG	TH	THY	TR	TRG	TRY	TT	TXH	TXX	YCY	YH	YM	YRB	YRQ	YXX	YZ	ZB
BT		3	0	0	0	2	2	0	1	1	0	0	0	5	0	1	0	1	0	0	1	0	0	1
DS	0		0	0	2	6	1	0	1	0	0	0	0	0	0	0	0	0	0	1	0	0	1	0
GS	2	3		0	0	2	2	0	1	1	0	0	0	0	0	4	0	0	0	0	2	0	0	1
HH	0	0	0		0	3	1	0	2	4	0	0	0	0	0	0	0	0	0	0	0	0	2	0
HM	0	0	0	0		0	0	0	0	2	0	0	0	0	0	0	0	0	0	0	2	0	0	0
HT	0	3	0	1	0		1	0	0	2	0	0	0	1	0	0	0	0	0	0	0	0	0	0
HXM	0	0	1	0	0	0		0	0	0	0	0	0	0	0	0	0	0	0	3	0	0	0	1
TG	2	1	0	0	0	4	3		1	3	0	0	0	2	0	3	1	3	3	1	1	3	2	1
TH	0	0	0	0	0	0	1	0		3	0	0	0	0	0	0	0	0	0	0	1	0	1	0
THY	0	0	0	0	0	0	0	1	0		0	0	0	0	0	0	0	0	0	0	0	0	0	0
TR	0	1	0	0	0	0	1	0	0	2		0	0	2	0	0	0	0	0	0	1	0	0	0
TRG	0	0	0	0	0	0	2	0	0	1	0		0	0	0	0	0	0	0	0	0	0	0	0
TRY	0	1	0	0	0	0	0	0	0	2	0	0		0	0	2	0	0	0	0	0	0	1	0
TT	0	0	0	0	0	0	0	0	0	0	0	0	0		0	0	0	0	0	0	0	0	0	0
TXH	0	0	0	0	1	1	0	0	0	0	0	0	0	3		2	0	0	1	0	0	0	2	0
TXX	0	1	0	0	0	0	2	0	0	0	0	0	0	2	0		0	1	0	0	0	0	0	0
YCY	0	0	0	0	1	0	0	0	0	2	0	0	0	0	0	0		1	1	0	0	2	0	0
YH	0	0	0	0	0	0	0	0	0	1	0	0	0	0	0	1	0		1	0	0	2	0	0
YM	0	0	0	0	0	0	0	1	1	0	0	0	0	0	0	0	0	0		0	0	0	0	0
YRB	2	2	0	5	2	4	6	2	0	0	0	0	0	2	0	1	0	3	1		2	8	2	5
YRQ	0	0	0	0	2	0	0	0	0	0	0	0	0	0	0	0	0	1	0	0		0	0	0
YXX	0	0	0	2	0	2	0	0	1	2	0	0	0	0	0	2	0	0	2	0	0		0	0
YZ	0	0	0	0	0	0	0	0	0	0	0	0	0	0	0	0	0	0	0	0	0	0		0
ZB	0	1	1	0	0	1	1	0	0	2	0	0	0	2	0	0	0	0	0	0	0	0	0	

Supplementary Table S5. Interaction matrix for Tibetan macaque agonistic competition data.

	BT	DS	GS	HH	HM	HT	HXM	TG	TH	THY	TR	TRG	TRY	TT	TXH	TXX	YCY	YH	YM	YRB	YRQ	YXX	YZ	ZB
BT		1	0	0	0	0	1	0	1	2	0	0	0	4	3	1	0	0	0	0	1	0	0	0
DS	0		0	0	0	1	0	0	0	0	0	0	1	0	0	0	0	0	0	0	0	0	0	0
GS	1	1		0	0	0	0	0	2	0	0	0	2	0	2	1	0	0	0	0	2	0	1	0
HH	0	0	0		0	1	0	0	1	1	0	0	1	2	0	0	0	0	0	0	0	0	2	0
HM	0	0	0	0		0	0	0	0	2	0	0	0	0	0	0	0	0	0	0	0	0	0	0
HT	0	1	0	0	0		1	0	0	2	0	0	0	1	1	0	0	0	0	0	0	0	0	0
HXM	0	0	0	0	0	0		0	0	0	0	0	0	0	0	0	0	0	0	0	0	0	0	0
TG	1	0	0	0	0	0	2		1	2	0	0	0	3	0	0	1	1	1	1	2	5	1	2
TH	0	0	0	0	0	0	1	0		0	0	0	2	0	0	0	0	0	0	1	1	0	1	0
THY	0	0	0	0	0	0	0	0	0		0	0	0	0	0	1	0	0	0	0	0	0	0	0
TR	0	1	0	0	0	0	1	0	0	1		1	1	1	0	0	0	0	0	1	1	0	1	0
TRG	0	0	0	0	0	0	1	0	0	1	0		0	0	0	0	0	0	0	0	0	0	0	0
TRY	0	1	0	0	0	0	0	0	0	2	0	0		0	0	0	1	0	0	1	0	1	1	1
TT	0	0	0	0	0	0	0	0	0	0	0	0	0		0	0	0	0	0	0	0	0	0	0
TXH	0	0	0	0	0	0	0	0	0	0	0	0	2	0		0	0	0	0	0	0	1	2	0
TXX	0	1	0	0	0	0	1	0	0	0	0	0	4	1	0		0	0	0	0	0	0	0	0
YCY	0	0	0	0	0	0	0	0	0	1	1	0	2	1	0	1		0	0	1	0	0	0	1
YH	0	0	0	0	0	0	0	1	0	1	0	0	0	1	0	2	1		1	0	2	2	0	0
YM	0	0	0	0	0	0	0	0	1	0	0	0	3	0	0	0	0	0		0	0	0	0	0
YRB	2	2	0	5	0	0	4	0	0	0	3	0	0	1	2	1	0	2	1		1	4	2	1
YRQ	0	0	0	0	0	0	0	0	0	0	0	0	0	0	0	0	0	0	0	0		0	0	0
YXX	0	0	0	3	0	0	0	0	1	2	0	0	4	0	2	3	4	0	1	2	0		1	1
YZ	0	0	0	0	0	0	0	0	0	0	0	0	0	0	0	0	0	0	0	0	0	0		0
ZB	0	0	0	1	0	1	0	1	0	0	0	0	0	1	0	0	0	0	0	0	0	0	0	

Supplementary Table S6. Interaction matrix for Tibetan macaque lack of agonism data
